# HIV-induced membraneless organelles orchestrate post-nuclear entry steps

**DOI:** 10.1093/jmcb/mjac060

**Published:** 2022-10-31

**Authors:** Viviana Scoca, Renaud Morin, Maxence Collard, Jean-Yves Tinevez, Francesca Di Nunzio

**Affiliations:** Advanced Molecular Virology Unit, Department of Virology, Institut Pasteur, Université Paris Cité, 75015 Paris, France; Imactiv-3D, 1 Place Pierre Potier, 31106 Toulouse, France; Advanced Molecular Virology Unit, Department of Virology, Institut Pasteur, Université Paris Cité, 75015 Paris, France; Image Analysis Hub/C2RT, Institut Pasteur, Université Paris Cité, 75015 Paris, France; Advanced Molecular Virology Unit, Department of Virology, Institut Pasteur, Université Paris Cité, 75015 Paris, France

**Keywords:** HIV, nuclear condensates, nuclear remodelling, phase separation, reverse transcription

## Abstract

HIV integration occurs in chromatin sites that favor the release of high levels of viral progeny; alternatively, the virus is also able to discreetly coexist with the host. The viral infection perturbs the cellular environment inducing the remodelling of the nuclear landscape. Indeed, HIV-1 triggers the nuclear clustering of the host factor CPSF6, but the underlying mechanism is poorly understood. Our data indicate that HIV usurps a recently discovered biological phenomenon, called liquid–liquid phase separation, to hijack the host cell. We observed CPSF6 clusters as part of HIV-induced membraneless organelles (HIV-1 MLOs) in macrophages, one of the main HIV target cell types. We describe that HIV-1 MLOs follow phase-separation rules and represent functional biomolecular condensates. We highlight HIV-1 MLOs as hubs of nuclear reverse transcription, while the double-stranded viral DNA, once formed, rapidly migrates outside these structures. Transcription-competent proviruses localize outside but near HIV-1 MLOs in LEDGF-abundant regions, known to be active chromatin sites. Therefore, HIV-1 MLOs orchestrate viral events prior to the integration step and create a favorable environment for the viral replication. This study uncovers single functional host–viral complexes in their nuclear landscape, which is markedly restructured by HIV-1.

## Introduction

Immediately after fusion at the plasma membrane, HIV-1 cores are released into the cytoplasm and move towards the nucleus, while the viral RNA (vRNA) genome begins the process of reverse transcription into double-stranded viral DNA (ds vDNA) (Di Nunzio, 2013; Campbell and Hope, 2015; Blanco-Rodriguez and Di Nunzio, 2021; Scoca and Di Nunzio, 2021a). Once imported in the nucleus, the interplay between the HIV-1 DNA genome and the host chromatin compartment is crucial for the fate of the virus–host coexistence. Recent discoveries in early steps of HIV life cycle (Dharan et al., 2020; Blanco-Rodriguez et al., 2020; Burdick et al., 2020; Selyutina et al., 2020; Guedan et al., 2021; Li et al., 2021; Rensen et al., 2021; Zila et al., 2021) revise the commonly accepted models of uncoating (loss of the viral capsid) and reverse transcription as exclusively cytoplasmic processes (Farnet and Haseltine, 1991; Suzuki and Craigie, 2007). It has been found that HIV prompts clusters of the cleavage and polyadenylation specificity factor subunit 6 (CPSF6) proteins outside the paraspeckles but colocalizing with some nuclear speckle (NS) factors (Francis et al., 2020; Rensen et al., 2021). However, the nature, behavior, and underlying mechanism behind the establishment of these organelles are still unknown.

Here, we distinguish the topological duplicity of the post-nuclear viral entry phases: the early and the late. Early post-nuclear entry steps involve the formation of the HIV-induced membraneless organelles (HIV-1 MLOs), composed of CPSF6 clusters enlarged with viral factors. Late post-nuclear entry steps are characterized by the completion of ds vDNA synthesis and integration. The final products of the reverse transcription process are recruited into the pre-integration complex (PIC) eventually for viral integration and transcription. The vDNA nuclear dynamics and its surrounding chromatin landscape dictate the evolution of HIV infection (Sharkey et al., 2000, 2011; Maldarelli, 2016). However, the mechanisms behind the fate of the proviral DNA in the nucleus remain unclear (Olson et al., 2019; Liu et al., 2020). These are challenging to study, also due to the limits imposed by available technologies. Real-time imaging approaches can provide new insight into unprecedented spatial information in single cells by tracking individual viruses.

Here, we perform single-cell studies. We describe early post-nuclear entry steps in which viral infection enhances the phase separation of CPSF6, building HIV-1 MLOs. Our results emphasize post-nuclear entry steps as finely regulated by the interplay between viral and host components. Of note, CPSF6 contains intrinsically disordered mixed-charge domains, which are responsible for the formation of liquid condensates *in vitro* (Greig et al., 2020) and may account for the formation of HIV-1 MLOs in infected cells. MLOs are established through a recently discovered biological phenomenon called liquid–liquid phase separation (LLPS), in which molecules transition from a liquid state to a more solid state, similarly to oil and water demixing (Brangwynne et al., 2009; Banani et al., 2016; Alberti et al., 2019). Our data reveal that HIV-1 MLOs undergo the rules of biomolecular condensates, concentrating viral and host factors in the same niche and hosting nuclear reverse transcription.

For the study of late post-nuclear entry steps, we labelled the newly synthesized ds vDNA through the HIV-1 ANCHOR DNA tagging system (Blanco-Rodriguez et al., 2020). In the mature PIC, ds vDNA is the only form able to give rise to a functional infection. Through tracking the vDNA and transcriptional vRNA, we found that transcriptional viral foci are excluded but remain in the vicinity of HIV-1 MLOs. Our finds suggest the role of HIV-1 MLOs in creating a surrounding microenvironment favorable for viral replication. We provide new insights into how HIV reprograms and markedly restructures the nuclear environment to orchestrate viral replication steps.

## Results

### HIV-1 MLOs are hubs of viral reverse transcription

**Figure 1:**
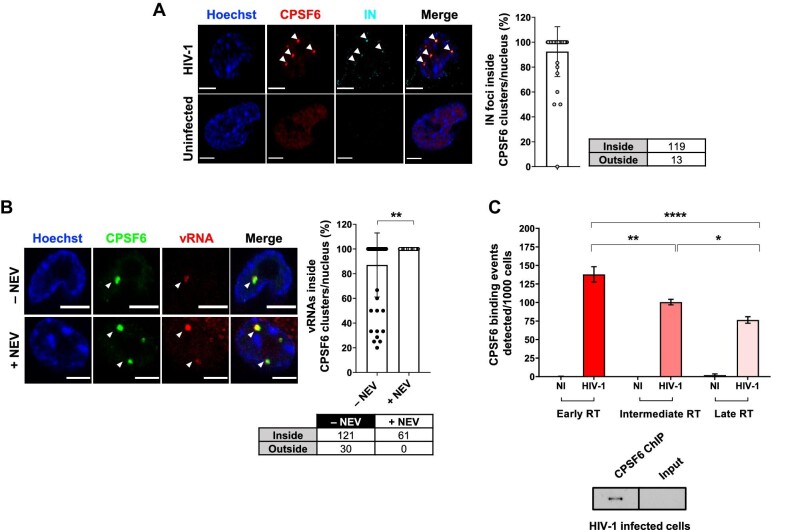
CPSF6 clusters host HIV-1 viral complexes and are hubs of nuclear reverse transcription. (**A**) Confocal microscopy images of HIV-1-infected THP-1 cells (MOI = 10, 30 h p.i.) compared to uninfected cells. The graph shows the percentage of IN associated with CPSF6 per nucleus ± SD (40 cells, 132 IN foci). Three independent experiments were performed. (**B**) Confocal microscopy images of immuno-RNA FISH in HIV-1-infected THP-1 cells (MOI = 20, 2 days p.i.) treated with or without NEV (10 µM). The graph shows the percentage of vRNA foci associated with CPSF6 per cell per condition ± SD (number of cells: 56 (–NEV), 34 (+NEV); number of vRNA foci: 151 (–NEV), 61 (+NEV)). Unpaired *t*-test, **P ≤ 0.01. Two independent experiments were performed. (**C**) ChIP of endogenous CPSF6 coupled to real-time qPCR of THP-1 cells infected with HIV-1 (MOI = 5, 2 days p.i.). Different reverse transcription products were amplified and normalized on the input ± SD. NI, non-infected; RT, reverse transcription. One-way ANOVA followed by Tukey's multiple comparison test, *P ≤ 0.05, **P ≤ 0.01, ****P ≤ 0.0001. On the bottom, western blot of CPSF6 of ChIP products in HIV-1-infected cells compared to the input. Scale bar, 5 µm.

Upon nuclear entry, the virus reprograms the nuclear localization of the host CPSF6 from being randomly dispersed in the nucleoplasm to discrete clusters (Figure 1A; Bejarano et al., 2019; Francis et al., 2020; Rensen et al., 2021). It has been suggested that nuclear entry of the viral capsid that interacts with CPSF6 (Price et al., 2014; Buffone et al., 2018) could drive the formation of CPSF6 clusters (Francis et al., 2020). Indeed, viruses carrying the capsid mutation N74D, unable to bind to CPSF6, do not prompt CPSF6 clustering formation (Supplementary Figure S1A; Selyutina et al., 2020). On the other hand, this phenomenon could also be prompted by the overexpression of CPSF6 (Chaudhuri et al., 2020) due to the viral infection.

Here, we characterize the HIV-1 post-nuclear step and its interplay with the host nuclear landscape in human macrophages. First, we evaluated the nuclear location of the viral endogenous integrase (IN) protein by immunolabelling the hemagglutinin (HA) tag fused to the C-terminal domain of IN (Petit et al., 2000; Blanco-Rodriguez et al., 2020). We confirmed that the majority of the viral IN proteins accumulate in foci occupied by CPSF6 clusters (Figure 1A; Supplementary Figure S2A; Francis et al., 2020; Muller et al., 2021; Rensen et al., 2021). To better define the relevance of these viral–host clusters, we performed an immuno-RNA single-molecule inexpensive fluorescence *in situ* hybridization (smiFISH), specific for HIV-1, and uncovered the presence of incoming vRNA inside CPSF6 clusters (Figure 1B). Upon the block of reverse transcription through nevirapine (NEV) treatment, we observed incoming vRNA genomes completely located inside CPSF6 clusters. Likewise, at 48 h post-infection (p.i.) under physiological infection conditions without drug treatment, vRNA foci could be detected inside CPSF6 clusters in most of the cells (Figure 1B). Only few cells showed a mixed population of vRNA foci inside and outside CPSF6 clusters, suggesting that the detected vRNAs can have a different nature (Figure 1B). Then, we explored the functional role of the coexistence of the incoming vRNA and IN proteins sequestered inside CPSF6 clusters. We performed chromatin immunoprecipitation (ChIP) of the endogenous CPSF6 protein followed by real-time quantitative polymerase chain reaction (qPCR) in HIV-1-infected and uninfected macrophage-like cells (THP-1) (Figure 1C; Supplementary Figure S1B). Early, intermediate, and late reverse transcription products were amplified using appropriate primers. Different stages of reverse transcription products were all associated with CPSF6, suggesting a reverse transcription activity inside CPSF6 clusters (Figure 1C). Our results corroborate previous data showing EdU-labelled vDNAs clustering in nuclear niches in macrophages (Francis et al., 2020; Rensen et al., 2021), where nuclear reverse transcription activity was detected once the reverse transcriptase inhibitor NEV was removed (Rensen et al., 2021). Taken together, our new data combined with previous results indicate that CPSF6 clusters serve as hubs of nuclear reverse transcription. We called these hubs hosting viral and host factors ‘HIV-1 MLOs’.

### HIV-1 MLOs are phase-separated condensates

**Figure 2:**
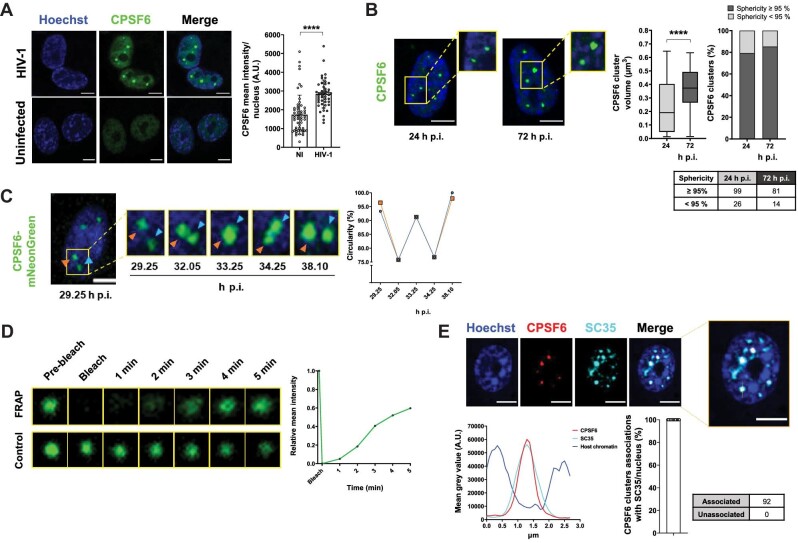
Phase-separation properties of HIV-1 MLOs. (**A**) Confocal microscopy images of THP-1 cells infected with HIV-1 (MOI = 5, 24 h p.i.) compared to uninfected cells. On the right, mean intensity of CPSF6 per nucleus ± SD (number of cells in the NI group = 49, number of cells in the HIV-1-infected group = 55). Unpaired *t*-test, ****P ≤ 0.0001. Two independent experiments were performed. (**B**) THP-1 cells were infected with HIV-1 (MOI = 5) and treated with NEV (10 µM). Confocal microscopy images of these cells at different time post-infection were compared (24 h p.i. vs. 72 h p.i). On the right, box plots of the volume (median: ∼0.19 vs. ∼0.37 µm^3^) and percentages of CPSF6 clusters that show ≥95% or <95% sphericity value (number of cells: 125 (24 h p.i.), 95 (72 h p.i)). Unpaired *t*-test, ****P ≤ 0.0001. Results of two independent experiments were analyzed from 3D acquisitions. (**C**) Frames extracted from a 10-h time-lapse microscopy in 2D (Supplementary Video S1) of THP-1 cells expressing CPSF6 mNeonGreen infected with HIV-1 (MOI = 10). The graph shows the circularity (normalized ratio between the contour and interior expressed as a percentage) along the MLO fusion–fission event. Representative of six independent experiments. (**D**) Frames extracted from a FRAP time-lapse microscopy (Supplementary Video S2) in THP-1 cells expressing CPSF6 mNeonGreen infected with HIV-1 (MOI = 10, 3 days p.i.). Representative of three independent experiments. The graph shows the recovery of the signal curve. Pre-bleach signal is set to 1 and bleach signal is set to 0. (**E**) Confocal microscopy images of THP-1 cells infected with HIV-1 (MOI = 10, 3 days p.i.). On the bottom, graphs show the intensity profile of Hoechst, CPSF6, and SC35 signals along the segment crossing the MLO (yellow in top right image) and the percentage of CPSF6 colocalizing with SC35 per nucleus ± SD. Results of two independent experiments were analyzed from 3D acquisitions. Scale bar, 5 µm.

Next, we investigated the phenomenon underlying the establishment of HIV-1 MLOs. MLOs allow the fine regulation of complex functions in a spatiotemporal manner (Banani et al., 2016; Fare et al., 2021). MLOs are generated by a newly characterized LLPS process that can be driven by the spontaneous assembly of intrinsically disordered regions (IDRs) present in some proteins (Li et al., 2012). Of note, CPSF6 contains disordered regions, such as arginine-enriched mixed-charge domains that have been evoked as responsible for the phase-separation features of this protein (Greig et al., 2020). To investigate whether LLPS aids the virus to navigate into the nuclear space through CPSF6, we performed a series of experiments. First, we observed an increase in CPSF6 intensity exclusively in the nucleus of infected cells (Figure 2A), in line with a recent observation that HIV-1 induces an increase of CPSF6 proteins (Chaudhuri et al., 2020). This observation suggests that CPSF6 during viral infection undergoes LLPS, known as a concentration-dependent phenomenon (Alberti et al., 2019). We also observed a reduction of CPSF6 clusters in infected cells treated with 1,6-hexanediol. This drug dissolves protein droplets driven by IDRs, commonly used to test LLPS *in vitro*. However, these findings should be viewed with caution. Indeed, our experiment also showed toxicity (nuclear morphology drastically affected) due to this compound (Supplementary Figure S2H), as previously reported (Wheeler et al., 2016). Second, we found that CPSF6 clusters increased the volume twice along the time post-infection (72 h p.i. vs. 24 h p.i.) and they were spherical (Figure 2B), typical shape of liquid droplets (Brangwynne et al., 2011; Strom and Brangwynne, 2019). The mean intensity of CPSF6 signal positively correlated with the mean intensity of IN proteins in the cluster (Pearson's r = 0.8563; Supplementary Figure S2A). Third, to evaluate their dynamics, we live-tracked HIV-1 MLOs. We used differentiated THP-1 cells stably expressing CPSF6 fused to mNeonGreen (Zhong et al., 2021). Of note, in almost all cases, the endogenous-only CPSF6 clusters were occupied by the exogenous fluorescent proteins, and CPSF6 mNeonGreen clusters carried viral components, such as IN (Supplementary Figure S2B). Thus, we performed time-lapse epifluorescence microscopy at 5-min intervals to capture fusion events (Figure 2C; Supplementary Video S1). We could appreciate the high dynamism of the HIV-1 MLOs, which kept restoring their circular shape (Figure 2C; Supplementary Figure S2C). Along our videos acquired at 24–72 h p.i., HIV-1 MLOs constantly evolved and performed fusion and fission (Figure 2B and C; Supplementary Figure S2C–E), which characterize LLPS condensates. Next, to evaluate whether HIV-1 MLOs are characterized by high mobility of the proteins essentially depending on diffusion, we performed fluorescence recovery after photobleaching (FRAP). Free CPSF6 proteins in the nucleoplasm showed high diffusion (Supplementary Figure S2F). In the context of infected cells, we observed that, after the laser irradiation (200 µs/px), the florescence of CPSF6 cluster started to recover at ∼2 min, indicating the exchange of fluorescent CPSF6 molecules inside and outside the MLO (Figure 2D; Supplementary Video S2), in contrast to the viral IN cluster that did not recover after bleaching (Supplementary Figure S2G). Nevertheless, this experiment is suggestive of LLPS behavior, in line with other fluorescent recoveries detected for other factors that follow LLPS properties in NSs (Supplementary Figure S2I; Marzahn et al., 2016; Taylor et al., 2019; Hong et al., 2020). We also observed that CPSF6 clusters fully colocalized with the NS marker SC35 (Figure 2E), in line with previous studies (Francis et al., 2020; Rensen et al., 2021). CPSF6 IDRs are the major players to promote NS residence (Greig et al., 2020). We remarked that CPSF6 and SC35 signals mainly occupy interchromatin space with low-level Hoechst stain (Figure 2E). We also confirmed that SC35 shows rapid recover of the fluorescence signal after photobleaching (Supplementary Figure S2I; Marzahn et al., 2016).

Our results inscribe HIV-1 MLOs in the plethora of biomolecular condensates (Alberti et al., 2019). Our observations suggest that HIV-1 MLOs assemble through a multistep process initiated by CPSF6–virus complexes and then assembling via phase separation. The condensates seem to exhibit a central stable structure decorated by highly dynamic CPSF6 proteins that exchange with the surrounding environment.

### The newly synthesized ds vDNA separates from HIV-1 MLOs

**Figure 3:**
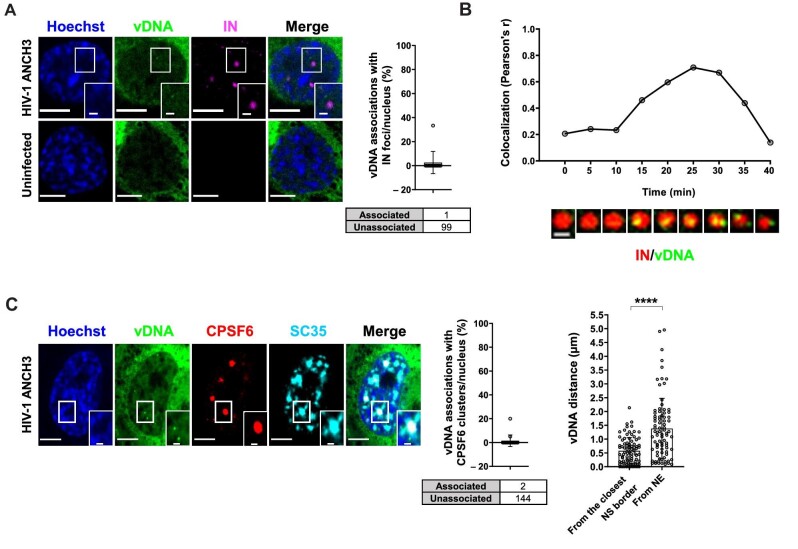
Complete reverse transcribed products are released from HIV-1 MLOs. (**A**) Confocal images of THP-1 cells expressing OR-GFP infected with HIV-1 ANCH3 (MOI = 10, 30 h p.i.) compared to uninfected cells. The graph shows the percentage of vDNAs associated with IN per nucleus ± SD (13 cells, 100 vDNAs). Results of two independent experiments were analyzed from 3D acquisitions. (**B**) Cropped frames from a time-lapse microscopy (Supplementary Video S3) of vDNA (green) and IN (red) dynamics in THP-1 cells (HIV-1 ANCH3 GIR, 80 h p.i.). Pearson's correlation of the two signals per 2D frame along the time. Representative of three independent experiments. Scale bar, 1 µm. (**C**) Confocal images of THP-1 cells expressing OR-GFP infected with HIV-1 ANCH3 (MOI = 10, 3 days p.i.). The graphs display the percentage of vDNA associated with CPSF6 clusters per nucleus ± SD (17 cells, 146 vDNAs) and the distance of each vDNA from the closest NE point or SC35-marked NS border ± SD (7 cells, 83 vDNAs). Results of two biological replicates were analyzed from 3D aquisitions. Unpaired *t*-test, ****P ≤ 0.0001. Scale bar, 5 µm.

To decipher the nuclear location of the newly synthesized ds vDNA, we benefited from HIV-1 ANCHOR system (Supplementary Figure S3A; Blanco-Rodriguez et al., 2020). Once fully reverse-transcribed, the HIV-1 genome carrying the bacterial sequence ANCH3 is specifically recognized by OR-GFP protein, which is expressed in the target cells, genetically modified by the lentiviral vector (LV) OR-GFP (Supplementary Figure S3A; Blanco-Rodriguez et al., 2020). The accumulation of OR-GFP, which is a modified version of the bacterial ParB protein (Graham et al., 2014; Sanchez et al., 2015), on the ANCH3 sequence generated bright nuclear signals (Figure 3A and B; Supplementary Figure S3B and C). OR protein binds exclusively to ds DNA (Saad et al., 2014). We cloned ANCH3 in the Nef gene, and HIV-1 ANCHOR system exclusively detected the final products of the reverse transcription, because ANCH3 sequence is one of the last sequences to be converted into ds vDNA (Supplementary Figure S3A). Thus, the system tracks the vDNA forms that will potentially be engaged in the PIC. Unlike previous studies based on viral protein tracking (Lelek et al., 2012; Chin et al., 2015; Burdick et al., 2017, 2020; Francis and Melikyan, 2018; Francis et al., 2020), we are now able to directly visualize individual nuclear vDNA (GFP spots) in fixed and live cells. The high specificity of HIV-1 ANCHOR system to detect the newly synthesized vDNA is shown by the correlation between the nuclear GFP puncta and the multiplicity of infection (MOI) used (Supplementary Figure S3B). In addition, the reverse transcriptase inhibitor, NEV, completely prevented the detection of vDNA (Supplementary Figure S3B). We also assessed whether HIV-1 ANCHOR system can detect both episomal and integrated forms of vDNAs. Indeed, this labelling system could reveal intranuclear GFP spots in infected cells treated with an inhibitor of integration, raltegravir (RAL), or by using an integration-defective virus (HIV-1 ANCH3 IN_D116A_) as confirmed by qPCR (Supplementary Figure S3C). Importantly, we also observed that HIV-1 ANCHOR system was sufficiently sensitive to allow the detection of a single provirus, as shown by HIV integration results and single-cell imaging of a selected clone (Supplementary Figure S3D).

Intriguingly, when we labelled late reverse transcripts with respect to the IN foci hosted in HIV-1 MLOs, we found a complete separation, since their association was extremely rare (Figure 3A). The HIV-1 IN–vDNA nuclear dynamics represent a long-standing and debated topic. There are, indeed, multiple challenges due to the short-time nature of the event or the technical limitations of vDNA and IN visualization. Thus, we performed live imaging, asking whether we could pinpoint the dynamic evolution of this separation, by coupling HIV-1 ANCHOR system with the Gag-IN-Ruby (GIR) virus (a generous gift from Edward Campbell) (Figure 3B; Supplementary Video S3; Hulme et al., 2015; Dharan et al., 2016). The virus is produced using a viral genome carrying ANCH3 sequence together with the GIR plasmid, incorporating fluorescent IN proteins in the viral particle. Performing multiple videos in THP-1 cells during a non-synchronized infection, we found IN foci (marking HIV-1 MLOs) in which several vDNA puncta arose before their separation (Figure 3B; Supplementary Video S3). We analyzed the association of the two signals, which rapidly decreased (Figure 3B). According to these data, final products of reverse transcription form inside HIV-1 MLOs. Of note, in fixed cells, we could label the nascent vDNAs via EdU incorporation that remained mostly associated with CPSF6 clusters, as opposed to the late reverse transcription products mainly detected outside CPSF6 clusters (Supplementary Figure S3E). The vDNA/IN separation data in fixed and live cells indicated that final products of the reverse transcription are released from HIV-1 MLO sites once synthesized, in line with a recent study (Muller et al., 2021). At 3 days p.i., nearly all double-stranded late reverse transcripts were excluded from HIV-1 MLO cores (Figure 3C), as also suggested from the separation of the vDNA from the IN puncta (Figure 3A and B). The majority of the viral IN proteins visible by confocal microscopy remained sequestered in CPSF6 clusters (Figure 1A), likely part of the multiple viruses retained in these organelles. Overall, our results indicate that IN proteins that are part of individual HIV PIC can be hardly visualized. The limit of resolution of a conventional light microscope can account for the inability to track a single HIV PIC, even if IN proteins form high-order structures in an individual PIC (Hare et al., 2009; Ballandras-Colas et al., 2017; Passos et al., 2017, 2020).

Next, we investigated the nuclear space distribution of the final products of reverse transcription. We computed the 3D distances of the ds vDNAs from the boundary of the closest NS or nuclear envelope (NE) and found that the vDNAs located closer to the NS than to the NE, at a distance ∼0.57 µm on average (Figure 3C). Therefore, our results show that the vDNAs labelled by HIV-1 ANCHOR system are mainly excluded from HIV-1 MLOs.

### HIV-1 active proviruses locate outside HIV-1 MLOs

**Figure 4:**
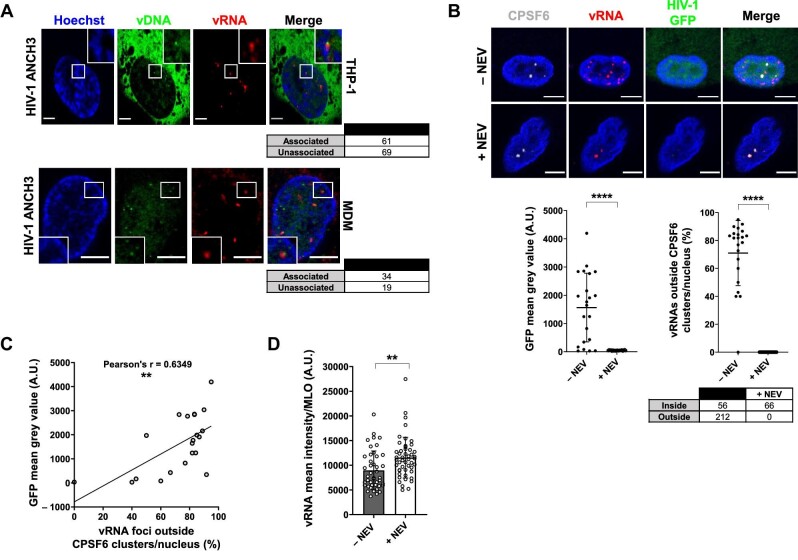
Active proviruses are excluded from HIV-1 MLOs. (**A**) Confocal images of THP-1 cells and primary MDMs expressing OR-GFP infected with HIV-1 ANCH3 (THP-1: MOI = 20, 3 days p.i.; MDMs: MOI = 40, 4 days p.i.). Analysis was performed from 3D acquisitions. (**B**) Confocal images of immuno-RNA FISH in primary MDMs infected with HIV-1 GFP (MOI = 20) and treated with or without NEV (10 µM) 2–3 days p.i. The scatter plots show the GFP intensity per cell ± SD and the percentage of vRNA foci outside CPSF6 clusters per nucleus ± SD (number of cells: 22 (–NEV), 20 (+NEV); number of vRNA foci: 268 (–NEV), 66 (+NEV)); Unpaired *t*-test, ****P ≤ 0.0001. (**C**) Correlation of GFP intensity and the percentage of vRNA foci excluded from CPSF6 clusters in HIV-1-infected cells (22 cells). Pearson's r coefficient, **P ≤ 0.01. (**D**) vRNA intensity per CPSF6 cluster per condition ± SD; unpaired *t*-test, **P ≤ 0.01. All the analyses were performed in 3D from two independent experiments. Scale bar, 5 µm.

Taking in consideration that ds vDNAs are found outside HIV-1 MLOs (Figure 3A–C), we wondered about the location of viral transcription sites that allow the viral replication. Thus, we coupled HIV-1 ANCHOR system with RNA FISH to co-detect the vDNA and the vRNA. The vDNA/vRNA association analysis highlights the transcription sites of the virus in both differentiated THP-1 cells and primary monocyte-derived macrophages (MDMs) (Figure 4A). Not all vDNAs were associated with a transcriptional focus, because the episomal forms are less actively transcribed than integrated vDNAs. However, some vDNAs colocalized with bright vRNA spots (RNA FISH), suggesting that these are active proviruses.

Next, we focused on the nuclear location of active proviruses in primary MDMs infected with HIV-1 GFP reporter virus to monitor viral expression (Figure 4B and C; Supplementary Figure S4A). Through immuno-RNA FISH, we were able to co-visualize CPSF6 clusters and HIV-1 vRNA as incoming genomes and viral transcription foci. We observed that at 3 days p.i., NEV-treated MDMs contained only incoming vRNAs sequestered in CPSF6 clusters (Figure 4B), similarly to THP-1 cells (Figure 1B). On the other hand, untreated cells presented a mixed population of vRNA foci inside and outside HIV-1 MLOs (Figure 4B). The percentage of excluded vRNA foci correlated with GFP expression level (Pearson's r ∼0.63), suggesting that these foci are actively transcribing proviruses (Figure 4B and C).

Moreover, we found that the mean intensity of the vRNA signal in HIV-1 MLOs (MLOs selected for sphericity > 95% as shown in Supplementary Figure S4B) was higher on average in NEV-treated cells compared to untreated cells (Figure 4D), likely due to an ongoing reverse transcription process (Figure 1C). The MLOs-excluded vRNA foci can be also tracked down by coupling HIV-1 ANCHOR system (vDNA detection) to immuno-RNA FISH (vRNA detection), indicating that the sites of viral transcription locate outside HIV-1 MLOs (Supplementary Figure S4C). These results are further supported by the decrease in number of excluded foci in RAL-treated infected cells (Supplementary Figure S4D), which did not show HIV-1 GFP reporter signal, and consistent with the notion that episomal forms transcribe much less than proviruses (Dupont et al., 2021).

**Figure 5:**
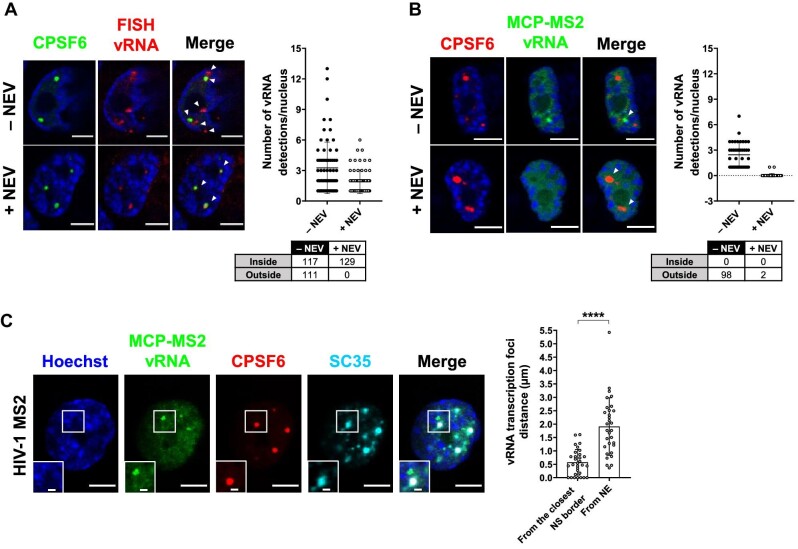
MCP-MS2 RNA labelling system detects transcription foci only. (**A**) Confocal images of immuno-RNA FISH in THP-1 cells infected with HIV-1 and treated with or without 10 µM NEV (MOI = 20, 3 days p.i.). The graph shows the number of vRNA per nucleus, and the table shows the amount of vRNA inside or outside CPSF6 clusters (number of cells: 70 (–NEV), 68 (+NEV)). Two independent experiments were performed. (**B**) Confocal images of THP-1 cells expressing MCP-GFP infected with HIV-1 ANCH3 MS2 and treated with or without 10 µM NEV (MOI = 2.5, 3 days p.i.). The graph shows the number of vRNA per nucleus, and the table shows the amount of vRNA inside or outside CPSF6 clusters (number of cells: 40 (–NEV), 44 (+NEV)). Three independent experiments were performed. (**C**) Confocal images of THP-1 cells expressing MCP-GFP infected with HIV-1 ANCH3 MS2 (MOI = 2.5, 3 days p.i.). The graph shows the distance of each RNA focus from the closest NE point or SC35-marked NS border ± SD (31 vRNAs, 8 cells). Results of two independent experiments were analyzed from 3D acquisitions. Unpaired *t*-test, ****P ≤ 0.0001. Scale bar, 5 µm. Inset, 1 µm.

The immuno-RNA FISH highlights both vRNA foci inside and outside the MLO (Figure 5A). On the contrary, when applying MCP-MS2 system (a kind gift from Edouard Bertrand) (Tantale et al., 2016), we were able to exclusively visualize vRNAs located outside HIV-1 MLOs. Upon the block of viral integration by using RAL, we could rarely detect vRNA foci, due to the low transcription level of episomal forms (Dupont et al., 2021), while in the absence of drug treatment, we could highlight several vRNA foci, indicating that these bright spots are active proviruses located outside CPSF6 clusters (Supplementary Figure S5A). Thus, MCP-MS2 system applied in the context of HIV exclusively detected viral transcription foci (Figure 5B; Supplementary Figure S5A). Besides, we observed that MCP-GFP was hindered from binding to the incoming vRNA and filtered out by HIV-1 MLOs. In fact, upon NEV treatment, no vRNA species were detected inside CPSF6 clusters (Figure 5B). The specificity of MCP-MS2 system in the binding of vRNA transcription foci could be due to the steric hindrance from the capsid that shields the vRNA genome (Li et al., 2021; Muller et al., 2021; Zila et al., 2021) or because MLOs can act as selective filters (Alberti et al., 2019). However, further investigations are needed to better characterize this phenomenon. In addition, this approach allows the live tracking of individual proviral transcription foci when coupled with HIV-1 ANCHOR technology (Supplementary Figure S5B). Overall, MCP-MS2 system is a valuable tool to study the interplay between active proviruses and the surrounding nuclear landscape. Thus, we used MCP-MS2 system to compute the 3D distances of viral transcription foci from the closest NS boundary or the NE. We observed that viral replication sites located in the NS-surrounding chromatin (on average ∼0.57 µm) (Figure 5C). These results, uploaded on bioRxiv in 2020 (https://doi.org/10.1101/2020.11.17.385567), have been confirmed by a recent study from another group (Burdick et al., 2022). It has been found by genomic approaches that HIV-1 favors integration in speckle-associated domains (Francis et al., 2020), known to be active chromatin loci (Kim et al., 2020). Our study allows to spatially distinguish and visualize individual vRNA transcription foci in a single cell. We are able to appreciate the nuclear topology of active proviruses.

### HIV-1 replication occurs in LEDGF-marked chromatin sites near HIV-1 MLOs in macrophage-like cells

**Figure 6:**
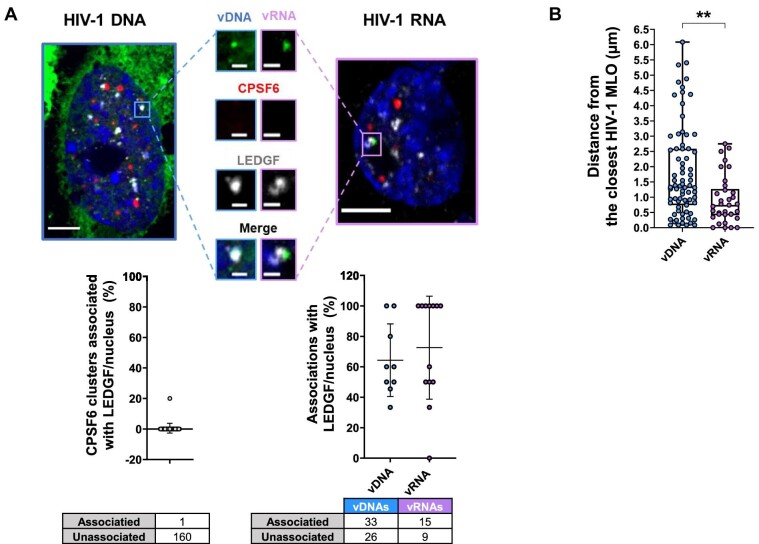
HIV-1 active proviruses locate in LEDGF-marked chromatin neighboring HIV-1 MLOs. (**A**) Confocal images of THP-1 cells expressing OR-GFP infected with HIV-1 ANCH3 (MOI = 10, 3 days p.i.) and expressing MCP-GFP infected with HIV-1 ANCH3 MS2 (MOI = 2.5, 3 days p.i.). On the bottom, the plots show the percentage of CPSF6 clusters associated with LEDGF per nucleus ± SD (38 cells, 161 CPSF6) and the percentage of vDNA or vRNA associated with LEDGF cluster per nucleus ± SD. Two independent experiments were performed. (**B**) Scatter plot shows the distance of vDNA or vRNA from the border of the closest HIV-1 MLO (SC35 + CPSF6) ± SD (75 vDNAs, 7 cells; 34 vRNAs, 8 cells). Unpaired *t*-test, **P ≤ 0.01. Results of two biological replicates were analyzed from 3D acquisitions. Scale bar, 5 µm. Inset, 1 µm.

We explored the nuclear landscape surrounding HIV-1 MLOs using HIV-1 ANCHOR and MCP-MS2 labelling systems. Lens epithelium-derived growth factor (LEDGF) (Ciuffi et al., 2005), a host partner of the viral IN (Cherepanov et al., 2003; Ciuffi et al., 2005; Emiliani et al., 2005; Llano et al., 2006; Shun et al., 2007, 2008; Hare et al., 2009; Ferris et al., 2010; Wang et al., 2014; Lelek et al., 2015), participates in HIV-1 integration site distribution. We observed that LEDGF formed clusters independently of viral infection in macrophage-like cells (THP-1) (Supplementary Figure S6). LEDGF is located in active chromatin regions, because its PWWP domain has been shown to interact with H3K36me3 (Pradeepa et al., 2012, 2014), which is a marker of euchromatin. This post-translational modification is enriched in HIV-1 integration sites (Ciuffi et al., 2005; Wang et al., 2009; Lelek et al., 2015; Vansant et al., 2020). In this study, we found LEDGF to be associated with the proviruses forming a complex visible by imaging and dissociated from CPSF6 clusters (Figure 6A). On average, ∼64% of vDNA and ∼73% of vRNA foci per nucleus were found in complex with LEDGF (Figure 6A). LEDGF is also known to interact with splicing factors (Singh et al., 2015). This agrees with our data showing vDNA and vRNA localized on average at ∼0.57 µm from the closest NS (Figures 3C and 5C). Our data support HIV-1-targeted active chromatin surrounding HIV-1 MLOs. More in detail, the foci of viral transcription locate at <1 µm from the closest HIV-1 MLO (CPSF6 + SC35), whereas the total vDNAs (episomal or integrated forms) are more distant, on average ∼1.8 µm (Figure 6B).

Overall, our results indicate that HIV-1 MLO-neighboring active chromatin regions, marked by LEDGF and in proximity of NS, favor HIV-1 integration, creating a suitable environment for viral replication.

## Discussion

Pathogens, such as HIV-1, able to integrate their genome into the host DNA have evolved to target chromatin both to favor the release of new progeny and to optimize their coexistence with the host (Bushman, 2003). Even though HIV-1 infection is characterized by the silent persistence of the virus in the host cell, HIV needs to rapidly generate high levels of virions during the acute phase, intuitively explaining the preference of active genes as integration targets (Schroder et al., 2002; Ciuffi et al., 2005; Di Nunzio et al., 2013; Lelek et al., 2015; Maldarelli, 2016).

Thus far, CPSF6 has been described as the crucial partner of HIV-1 capsid (Lee et al., 2010; Price et al., 2014; Buffone et al., 2018) and player in HIV-1 integration site selection (Sowd et al., 2016; Achuthan et al., 2018; Buffone et al., 2018). When CPSF6 was first discovered, a deletion mutant of CPSF6 unable to translocate in the nucleus was found as a potent inhibitor of HIV-1 infection (Lee et al., 2010). Here, we provide new insights on the properties of CPSF6 protein clusters induced by the infection, and we unravel their unexpected role in HIV-1 pre-integration steps in non-dividing cells, known to be an important cellular reservoir (Ganor et al., 2019; Kruize and Kootstra, 2019; Real and Bomsel, 2019; Veenhuis et al., 2021). CPSF6 proteins have been shown to cluster in NSs upon viral infection (Francis et al., 2020; Rensen et al., 2021). We observed that CPSF6 proteins form condensates that occupy the interchromatin space (Figure 2E) and host multiple viral components at early time post-infection (Figure 1A and B). These host–viral condensates form even in the absence of reverse transcription (Figure 1B), implying their relevance in early post-nuclear entry stages. Multiple viral EdU-labelled DNA copies strongly colocalize with CPSF6 clusters (Supplementary Figure S3E; Francis et al., 2020; Rensen et al., 2021). Indeed, ChIP of the endogenous CPSF6, followed by real-time qPCR in infected THP-1 cells (48 h p.i.), shows an abundance of early, intermediate, and late reverse transcription products. We also observed that the amount of the vRNA inside CPSF6 clusters decreases if there is a reverse transcription in progress compared with the vRNA sequestered in CPSF6 clusters in the presence of NEV (Figure 4D). These data are in line with our previous study that highlighted the existence of a nuclear reverse transcription, observed when NEV was removed, restoring the reverse transcription inside CPSF6 clusters (Rensen et al., 2021).

We named these nuclear viral/host niches ‘HIV-1 MLOs’ (Scoca and Di Nunzio, 2021b) and investigated their LLPS properties in the cell. CPSF6 contains intrinsically disordered mixed-charge domains, which are responsible for the formation of CPSF6 condensates *in vitro* (Greig et al., 2020). On the other hand, it seems that the binding of CPSF6 to the viral capsid is essential for CPSF6 clusters (Supplementary Figure S1A). Thus, we propose that CPSF6 interacts with viral complexes via the CA binding domain, and the phase-separation properties aid the virus to navigate inside the nuclear space to form HIV-1 MLOs. We show that HIV-1 MLOs match LLPS properties in the cells, being dynamic functional condensates and evolving along infection (Figure 2). Furthermore, the recovery of CPSF6 fluorescence after photobleaching indicates the importance of CPSF6 equilibrium maintenance in these structures (Figure 2D). CPSF6 phase separation with viral reverse transcription complexes may be induced as the response to the nucleoplasm perturbation by HIV, to find a new protein(s) balance. Of note, CPSF6 plays key roles in biological processes, such as in the mRNA processing. We observed an increased amount of this protein upon viral infection (Figure 2A), and this phenomenon could ensure a bimodal role of CPSF6, one for the biology of the host cell and the other for the viral replication.

Our data hinted that HIV-1 MLOs play a role in pre-integration steps. To characterize the later nuclear stages of the virus, we exploited HIV-1 ANCHOR system, which is the only DNA labelling approach permitting in fixed and live cells to track HIV-1 ds DNA, including proviruses (Figure 4A; Supplementary Figures S3D and S5B). The final reverse transcription products rarely colocalize with IN foci, leaving HIV-1 MLOs (Figure 3A–C). This may be due to the inability of confocal microscopy to detect the IN proteins that are part of the PIC or because the integration event occurs at the surface of the HIV-1 MLO to rapidly go further. We can live-track both proviral and episomal forms using HIV-1 ANCHOR system but we cannot distinguish them; however, we can follow the moment of their release from HIV-1 MLOs. At 3 days p.i., in non-dividing cells, the vDNA population is composed of both episomal and proviral forms. Next, confocal microscopy results indicate that the vDNAs labelled by HIV-1 ANCHOR system are excluded from MLOs, and the majority locate at ∼0.57 µm from the closest SC35-marked NS (Figure 3C). Likely, HIV exploits the induced CPSF6 clusters to generate PICs, which then depart from these interchromatin sites (Figure 3B). Notably, HIV-1 ANCHOR system is sensitive enough to enlighten a single provirus (Supplementary Figure S3D), and through coupling with virus-specific RNA smiFISH (Tsanov et al., 2016), it is possible to reveal transcriptionally active proviruses (Figure 4A). The RNA FISH in primary macrophages displays viral transcription foci excluded from CPSF6 clusters, as previously hinted by the vDNA location. The percentage of vRNA foci excluded from MLOs positively correlates with the intensity of HIV-1 GFP used as viral reporter gene (Figure 4B and C), indicating that the viral replication occurs outside HIV-1 MLOs, while the vRNA genomes reverse transcribe inside (Figures 1C, 3B, and 4D).

We finally focused on the nuclear landscape of viral expression, exploiting MCP-MS2-tagged viruses, which mark viral transcription foci only (Figure 5B; Supplementary Figure S5A and B). Viral transcription foci locate outside HIV-1 MLOs but in proximity of NSs (Figure 5C). LEDGF associates with highly spliced transcriptional units through its direct interaction with several splicing factors (Singh et al., 2015) and determines HIV-1 integration site distribution in active chromatin sites (Ciuffi et al., 2005; Shun et al., 2007; Ferris et al., 2010). However, a complex formed by the vDNA or vRNA and LEDGF was never highlighted by fluorescence imaging (Figure 6A). We uncovered that most of the cells present a high percentage of vRNA foci associated with LEDGF (Figure 6A), highlighting the active nature of HIV-1-targeted domains. Lastly, the vRNA transcription foci are closer to CPSF6 + SC35 condensates compared to the total ds vDNA nuclear forms, suggesting that the HIV-1 MLOs-neighboring regions are preferred hubs of viral replication (Figure 6B).

Taken together, our results show that CPSF6 phase-separates to generate speckles-enlarged MLOs prompted by the virus, likely, to usurp functions linked to speckle factors. We propose the following model: HIV-1 MLOs host viral complexes and their nuclear reverse transcription, but once the ds vDNA is synthesized, it is released from the MLOs. The NS-associated chromatin is a favorable environment for viral replication, since active proviruses locate at <1 µm from the closest HIV-1 MLOs (Figure 6B). Our study supports how the new single-cell-level approaches are pivotal in the study of functional nuclear dynamics of viruses to discriminate the cells susceptible to fuel viremia or that concur to viral persistence.

## Materials and methods

### Cell lines, primary cells, and generation of HIV-1 ANCH3 single-cell clones

THP-1 cells (obtained from ATCC) are immortalized monocytic cells, which, once seeded, differentiate into macrophage-like cells under phorbol 12-myristate 13-acetate (PMA) treatment (160 nM). HEK293T cells (obtained from ATCC) are human embryonic kidney cells used to produce LVs and HIV-1. HeLa P4R5 cells are β-galactosidase reporter cells expressing CD4, CXCR4, and CCR5 (Charneau et al., 1994). HeLa MCP-GFP cells stably express MCP-GFP bacterial fusion protein (Tantale et al., 2016). THP-1 cells were cultivated in RPMI 1640 medium supplemented with 10% fet al bovine serum (FBS) and 1% penicillin–streptomycin solution (100 U/ml). HEK293T and HeLa P4R5 cells were cultivated in Dulbecco’s modified Eagle medium supplemented with 10% FBS and 1% penicillin–streptomycin (100 U/ml). MDMs were obtained by PBMC buffy coat isolation from healthy donors’ blood via Etablissement Français du Sang, Paris, through density gradient centrifugation with Ficoll 400. The PBMCs were then incubated at 37°C for 2 h; next, non-adherent cells were washed away, and complete RPMI 1640 medium was supplemented with M-CSF (10 ng/ml). After 3 days, the medium was changed with RPMI without M-CSF and cells were left to differentiate for another 4 days before proceeding with experiments.

HIV-1 ANCH3 single-cell clone was generated from HeLa P4R5 cells infected with HIV-1 ANCH3 (MOI = 1). Cells were diluted to 1 cell/well in 96-well plates. Cell-clone colonies were tested for β-galactosidase expression to check viral infectivity (kit Roche #11758241001). Positive clones were transduced with the LV CMV-OR-GFP for the imaging of HIV-1 ANCH3 provirus. All cells were kept in incubator at 37°C and 5% CO_2_.

### Plasmids, LVs, and viral productions

HIV-1ΔEnvIN_HA_ΔNef plasmid encodes the ΔEnvHIV-1 LAI (BRU) viral genome where the IN protein is fused to the HA tag (Petit et al., 1999,  2000). HIV-1ΔEnvIN_HA_ΔNef ANCH3 (HIV-1 ANCH3) was obtained through ANCH3 insertion: ANCH3 sequence was cloned by PCR using the template plasmid pANCH3 as previously described in Blanco-Rodriguez et al. (2020). The ANCHOR^TM^ technology and sequences are exclusive property of NeoVirTech (Germier et al., 2017; Mariame et al., 2018). HIV-1ΔEnvIN_HA_(D116A)ΔNef ANCH3 (HIV-1 ANCH3 IN_D116A_) was obtained by insertional mutagenesis using the QuikChange II XL Site-Directed Mutagenesis kit (Agilent #200522) for IN mutation at D116. The LV plasmid pSICO-CPSF6-mNeonGreen encoding CPSF6 fluorescent fusion protein was a gift from Zandrea Ambrose (Addgene plasmid #167585; http://n2t.net/addgene:167585; RRID:Addgene_167585).

The LV SC35-mRuby plasmid was kindly provided to us by Roy Parker. HIV-1ΔEnvIN_HA_ΔNef ANCH3 MS2 plasmid was obtained by inserting the MS2 × 64 stem-loop sequence, from pMK123**-**MS2 × 64 stem-loop sequence plasmid (kindly provided by Edouard Bertrand) (Tantale et al., 2016), in HIV-1ΔEnvIN_HA_ΔNef ANCH3. HIV‐1ΔEnvΔNef IRES GFP plasmid encodes the ΔEnvHIV-1 NL4.3 GFP viral genome (Rensen et al., 2021). The LV plasmids CMV-OR-GFP and CMV-OR-SANTAKA were obtained by cloning OR-GFP/OR-SANTAKA cassette (plamids from NeoVirTech) in a pTripCMV (ΔU3) plasmid through PCR using restriction sites, *Age*I and *Sgr*DI**.**

LVs and HIV-1 viruses were produced by transient transfection of HEK293T cells through calcium chloride coprecipitation. Co-transfection was performed as follows: for LVs, 10 µg of transfer vector, 10 µg of packaging plasmid (gag-pol-tat-rev), and 2.5 µg of pHCMV-VSV-G envelope plasmid; for VSV-G HIV-1ΔEnv viruses, 10 µg HIV-1ΔEnv plasmid and 2.5 µg of pHCMV-VSV-G plasmid. LVs and viruses for transduction/infection of THP-1 cells and MDMs were produced in combination with 3 µg of SIV_MAC_ Vpx (Durand et al., 2013). HIV-1 ANCH3 IN_D116A_ has been also produced in combination with the GIR plasmid (Hulme et al., 2015; Dharan et al., 2016) for IN/vDNA live microscopy. After the collection of the supernatant 48 h post-transfection, lentiviral particles were concentrated by ultracentrifugation for 1 h at 22000 rpm at 4°C and stored at −80°C. LVs and viruses were titred by qPCR in HEK293T cells 3 days post-transduction.

### ChIP with qPCR

THP-1 cells (4×10^8^) infected or not with HIV-1 (MOI = 5) were fixed with 1% formaldehyde for 15 min and quenched with 0.125 M glycine, and then sent to Active Motif Services to be processed for ChIP–qPCR. In brief, chromatin was isolated by the addition of lysis buffer, followed by disruption with a Dounce homogenizer. Lysates were sonicated and the DNA sheared to an average length of 300–500 bp. Genomic DNA (input) was prepared by treating aliquots of chromatin with RNase, proteinase K, and heat for de-crosslinking, followed by ethanol precipitation. Pellets were resuspended and the resulting DNA was quantified on a Nanodrop spectrophotometer. Extrapolation to the original chromatin volume allowed quantitation of the total chromatin yield. An aliquot of chromatin (40 µg) was precleared with protein A agarose beads (Invitrogen). Genomic DNA regions of interest (ROIs) were isolated using 4 µg of antibody against CPSF6 (#NBP1-85676). Complexes were washed, eluted from the beads with sodium dodecyl sulfate buffer, and subjected to RNase and proteinase K treatment. Crosslinks were reversed by incubation overnight at 65°C, and ChIP DNA was purified by phenol–chloroform extraction and ethanol precipitation. The qPCR reactions were carried out in triplicate on specific genomic regions using SYBR Green SuperMix (BioRad). The resulting signals were normalized for primer efficiency by carrying out qPCR for each primer pair using input DNA. Different reverse transcription products were amplified using the following primers: early reverse transcription products: 5′-GCCTCAATAAAGCTTGCCTTGA-3′ and 5′-TGACTAAAAGGGTCTGAGGGATCT-3′; intermediate reverse transcription products: 5′-CTAGAACGATTCGCAGTTAATCCT-3′ and 5′-CTATCCTTTGATGCACACAATAGAG-3′; late reverse transcription products: 5′-TGTGTGCCCGTCTGTTGTGT-3′ and 5′-GAGTCCTGCGTCGAGAGAGC-3′. A group of control genes were also amplified to show the enrichment of HIV-1-specific DNA under the infected condition. De-crosslinked ChIP products were checked by western blot against CPSF6 (#NBP1-85676), loading 37.5 ng of DNA per sample.

### RNA FISH and immuno-RNA FISH

THP-1 cells were seeded on coverslips (12 mm #1, ThermoFisher) and differentiated with PMA (160 nM) for 48 h. For RNA FISH, the cells were transduced with OR-GFP LV (MOI = 0.5) and then infected with HIV-1 ANCH3 (MOI = 20) for 3 days. The medium was always supplemented with PMA. Primary MDMs were seeded before differentiation on coverslips and, after 7 days, transduced with OR-GFP LV (MOI = 10) and infected with HIV-1 ANCH3 (MOI = 40) for 4 days. Then, the cells were washed with Dulbecco's phosphate-buffered saline (DPBS), fixed with 4% paraformaldehyde (PFA) solution for 15 min, and incubated in 70% ethanol at –20°C at least overnight. Primary smiFISH probes have been designed against HIV-1 pol sequence and containing a shared readout sequence for secondary probe alignment. A total of 24 smiFISH probes (Supplementary Table S1) against HIV pol were designed with Oligostan (Tsanov et al., 2016) and purchased from Integrated DNA Technologies. Primary probes were pre-hybridized with a secondary FLAP probe conjugated to Cy5 fluorophore (Eurofins) through pairing with the readout sequence. Washes and hybridization were performed with Stellaris Buffers (WASH buffer A, WASH buffer B, hybridization buffer; LGC Biosearch Technologies), following the manufacturer protocol. Hybridization with the probe was carried out at 37°C in a dark humid chamber for 5 h.

For immuno-RNA FISH, THP-1 cells were seeded on coverslips, differentiated with PMA (160 nM) for 48 h, and then infected with HIV-1 (MOI and time points indicated in figure legends). NEV (10 µM) or RAL (20 µM) were used to block respectively DNA synthesis or integration. Primary MDMs were seeded before differentiation on coverslips and, after 7 days, infected with HIV-1 GFP reporter virus for 2–3 days.

Fixation was carried on in 4% PFA for 15 min and then the coverslips were incubated with a blocking/permeabilization solution (1% bovine serum albumin (BSA) in PBS, 2 mM vanadyl ribonucleoside complexes solution (Sigma #94742), and 0.3% Triton X-100) for 1 h. Then, the coverslips were incubated with anti-CPSF6 1:400 (#NBP1-85676) in blocking/permeabilization solution for 1 h, washed, and incubated for 45 min with anti-rabbit secondary antibody, at room temperature in a dark humid chamber. The coverslips were fixed again in 4% PFA for 10 min. Subsequent RNA FISH was carried out as mentioned before, except that the hybridization with the probes was carried on overnight.

Finally, all the coverslips were stained with Hoechst 33342 1:5000 (Invitrogen #H3570) for 5 min and mounted on glass slides (Star Frost) with Prolong Diamond Antifade Mountant (Life Technologies #P36970). Confocal microscopy was carried out with a Zeiss inverted LSM700 microscope, with a 63× objective (Plan Apochromat, oil immersion, NA = 1.4).

### Fluorescence microscopy

The cells were plated on coverslips (12 mm #1, ThermoFisher). THP-1 cells were differentiated with PMA (160 nM) for 48 h and then infected transduced/infected (MOI and time post-infection indicated in figure legends). For late reverse transcript or RNA visualization, the cells were transduced with OR-GFP LV (MOI = 0.5) or MCP-GFP LV (MOI = 2) and, 48 h later, infected with HIV-1 ANCH3 or HIV-1 ANCH3 MS2, respectively. The medium was always supplemented with PMA. For HIV-1 ANCH3 imaging in HeLa P4R5 cells, the cells were transduced with OR-GFP LV (MOI = 0.2) and, 24 h later, infected with HIV-1 ANCH3 or HIV-1 ANCH3 IN_D116A_. NEV (10 µM) or RAL (20 µM) was used to block respectively DNA synthesis or integration. On the day of fixation, the cells were washed with PBS and fixed with 4% PFA for 15 min. For protein staining, cells were treated with glycine 0.15% for 10 min, permeabilized with 0.5% Triton X‐100 for 30 min, and blocked with 1% BSA for 30 min. All antibody incubations were carried out at room temperature in a dark humid chamber, for 1 h with primary antibodies and 45 min with secondary antibodies. Washes between antibody incubations and antibody dilution were done in 1% BSA. Primary antibodies were diluted as follows: anti-HA 1:500 (Roche #11867423001), anti-CPSF6 1:400 (Novus Biologicals #NBP1-85676), anti-SC35 1:200 (Abcam #ab11826), and anti-LEDGF 1:200 (BD Bioscience #611715). Secondary antibodies used were: donkey anti-rabbit Cy3 1:1000 (Jackson Lab #711-165-152), goat anti-rabbit Alexa-488 1:300 (Invitrogen #A32731), goat anti-mouse Alexa-647 1:300 (Invitrogen #A21235), and donkey anti-rat Alexa-488 (Invitrogen #A21208) or goat anti-rat Alexa-647 (Invitrogen #A21247) 1:100 for IN-HA. For vDNA detection via EdU incorporation, the cells were infected in the presence of EdU (5 µM) for 3 days (adding new RPMI + EdU medium every day) and, after fixation, the click-chemistry was performed before immunostaining as described in Rensen et al. (2021). Finally, cells were stained with Hoechst 33342 1:10000 (Invitrogen #H3570) for 5 min. Coverslips were mounted on glass slides (Star Frost) with Prolong Diamond Antifade Mountant (Life Technologies #P36970). Confocal microscopy was carried out with a Zeiss LSM700 inverted microscope, with a 63× objective (Plan Apochromat, oil immersion, NA = 1.4).

### FRAP and time‐lapse microscopy

For all time-lapse studies, the cells were plated in a polymer-coverslip bottom µ-Dish 35 mm (ibidi #81156). THP-1 cells (2×10^6^) were differentiated for 48 h and then transduced with CPSF6 mNeonGreen LV (MOI = 0.01) for 3 days. Next, the cells were infected with HIV-1 and the live imaging was performed during 24–72 h p.i. For CPSF6 cluster fusion studies, CPSF6 mNeonGreen-positive cells were imaged every 5 min in 2D with a Biostation IM-Q (Nikon).

For FRAP experiments, the selected ROI was irradiated for 200 µs/pixel with 488 nm/561 nm laser and, after bleaching, the frames were acquired every 5–10 sec for 5 min through a Ti2E inverted microscope (Nikon), based on a CSU-W1 spinning-disk (Yokogawa), using a 60× objective (Plan Apochromat, oil immersion, NA = 1.4).

Experiments of live tracking of GIR and vDNA (OR-GFP) were performed in differentiated THP-1 cells transduced with OR-GFP LV (MOI = 5). Two days post-transduction, the cells were infected with HIV-1 ANCH3 GIR virus (MOI = 30). Different cells were imaged during 45–96 h p.i., every 5 min in 3D (stacks spacing 0.3 µm) with a Ti2E inverted microscope (Nikon), based on a CSU-W1 spinning-disk (Yokogawa), using a 60× objective (Plan Apochromat, oil immersion, NA = 1.4).

### Imaging analysis and statistics

All data were analyzed with GraphPad Prism 9 (GraphPad Software, www.graphpad.com), and statistic tests are indicated in figure legends. All images and videos were analyzed in Fiji (Schindelin et al., 2012) and Icy software 2.2.1.0 (de Chaumont et al., 2012). In particular, CPSF6 cluster segmentation was performed though HK-Means block in Icy (Dufour et al., 2008) followed by ROI statistics exportation, e.g. mean intensity, volume, area, sphericity/circularity defined as the normalized ratio between the contour and interior of the ROI, expressed as a percentage (100% for a circle or sphere). To measure the distance of vDNA/vRNA from SC35 or HIV-1 MLO, 3D confocal images were processed for multi-channel image splitting as well as vDNA and SC35 segmentation. The automated 3D segmentation of cell nuclei included a preliminary non-local means denoising step to cope with the strong signal heterogeneity, and the segmentation of SC35 was readjusted to low-contrast conditions. The 3D boundary-to-boundary distance was computed between each vDNA or vRNA spot and its closest Hoechst-marked NE border, SC35 speckle border, or the closest HIV-1 MLO (SC35 + CPSF6) border.

## Supplementary Material

mjac060_Supplemental_Files
