# Supplementary material for: HIV-induced membraneless organelles orchestrate post-nuclear entry steps
Source: J Mol Cell Biol. 2022 Oct 31;14(11):mjac060. doi: 10.1093/jmcb/mjac060 (PMC10117160; doi:10.1093/jmcb/mjac060)
Supplement: mjac060_Supplemental_Files [file mjac060_supplemental_files.zip › Supplementary Material and table.docx]

**Supplementary Material**

**Supplementary Figures**


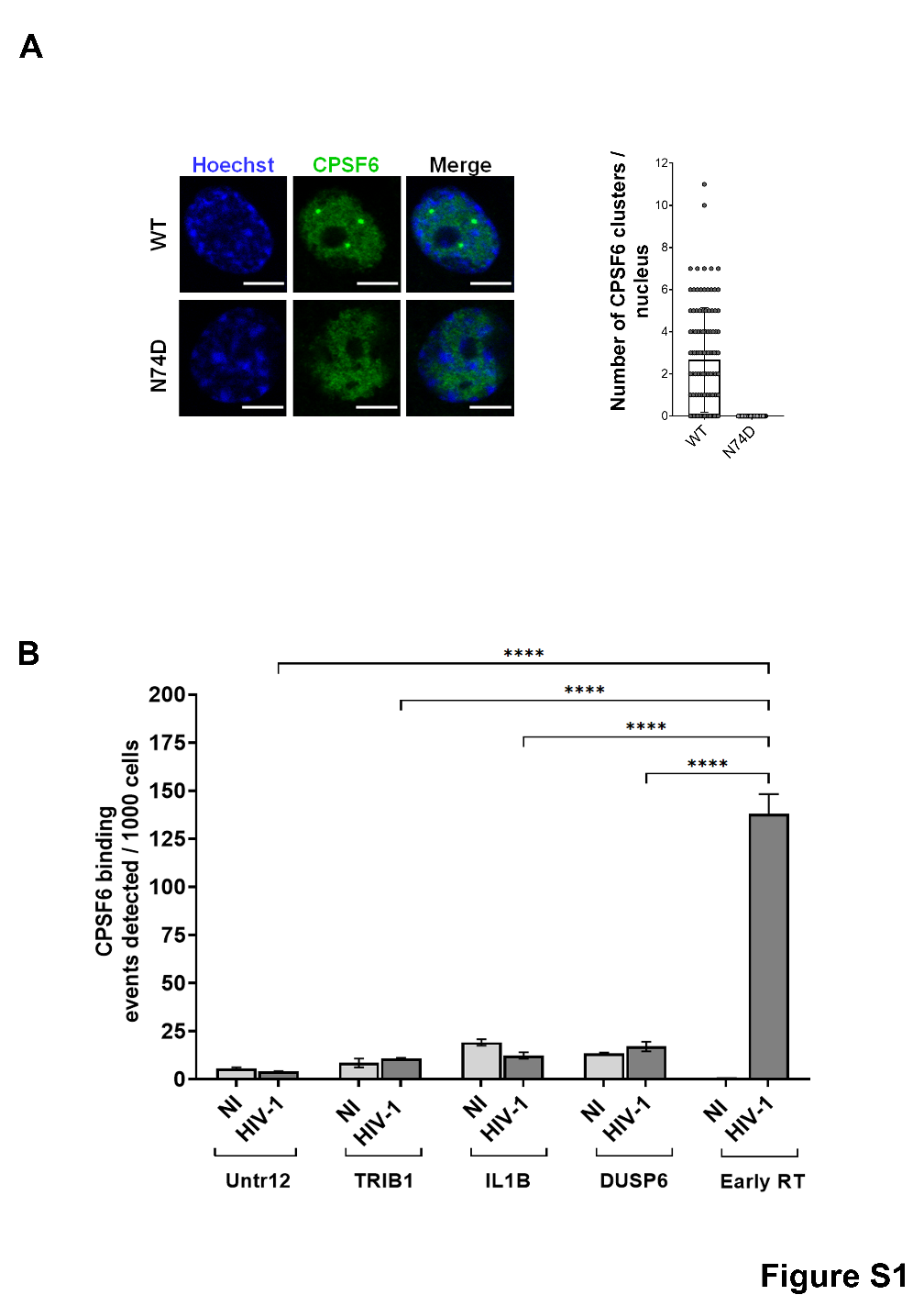


**Supplementary Figure S1. A)** Confocal microscopy images of THP-1 cells infected with CA WT and CA N74D HIV-1 (MOI 5, 3 days p.i.). On the right, CPSF6 clusters count per cell ± SD (n cells = 108, 120). Unpaired t test, ****=p ≤ 0.0001. **B)** The histogram plot shows the number of copies ± SD of random genes in CPSF6 ChIP normalized for the input, compared to HIV-1 early reverse transcripts (RT) also normalized for the input (Figure 1C). THP-1 cells infected with HIV-1 (MOI 5), 2 days p.i. One-way ANOVA followed by Tukey’s multiple comparison test, ****=p ≤ 0.0001.


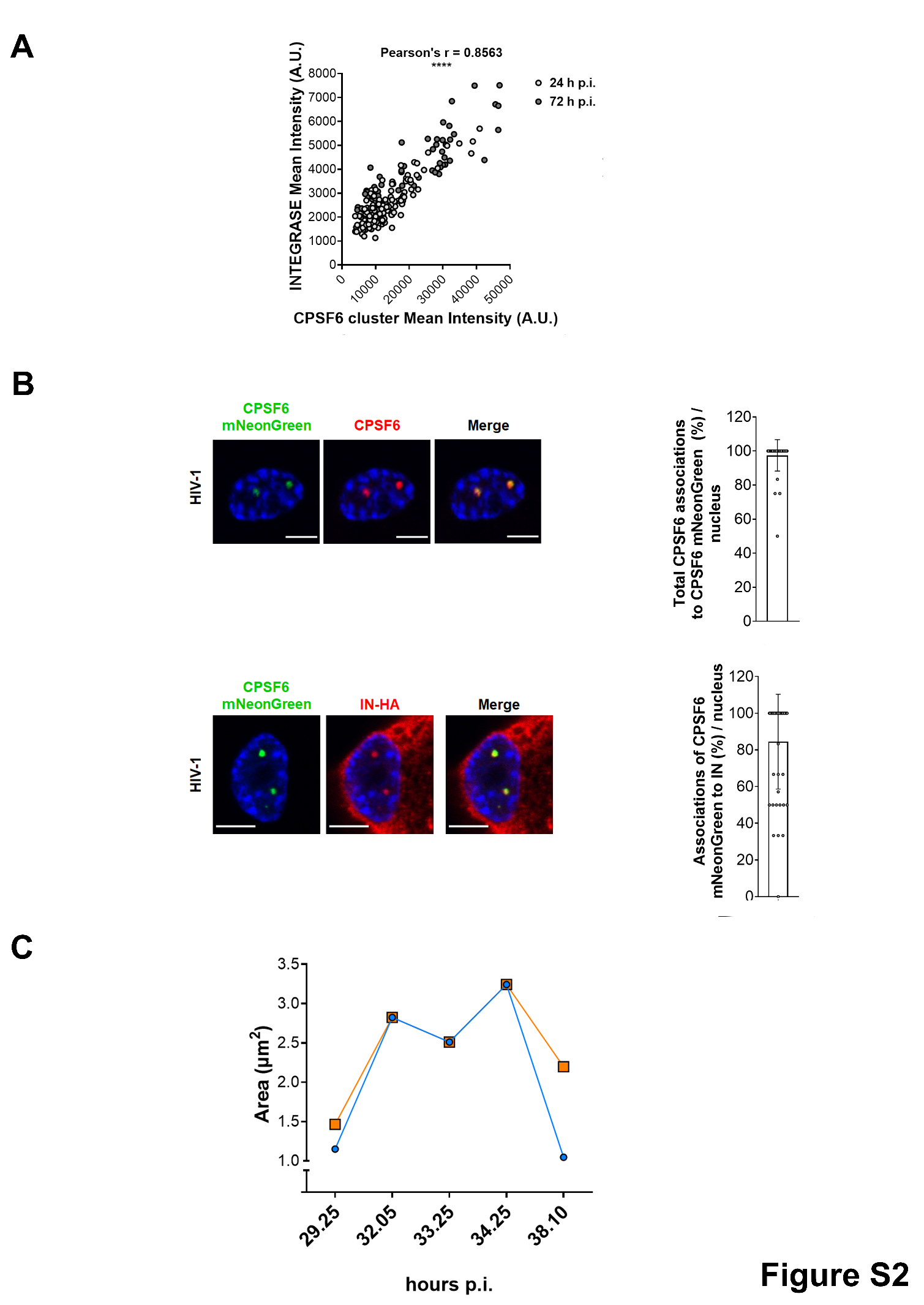

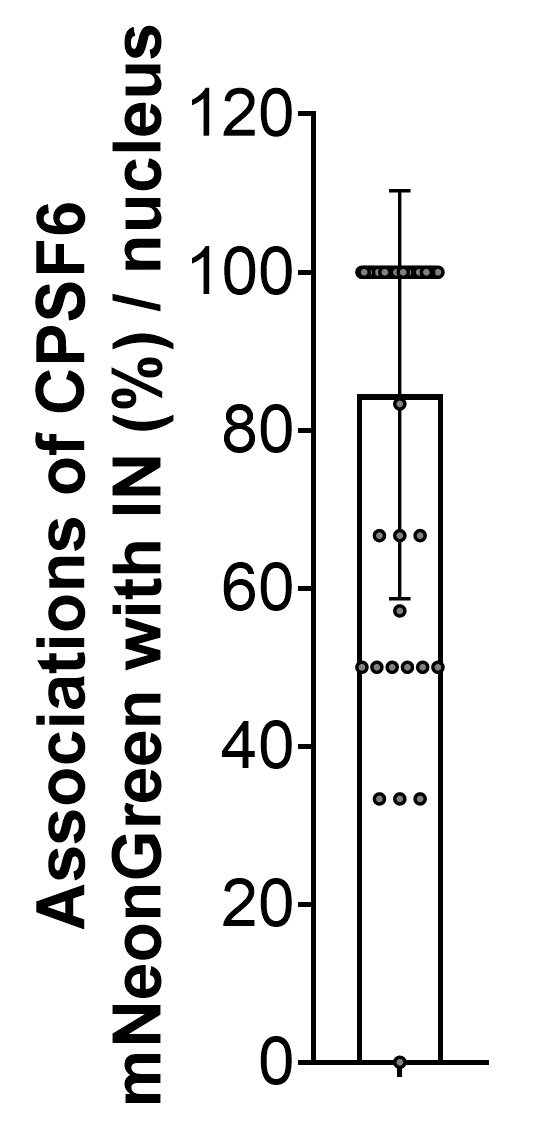

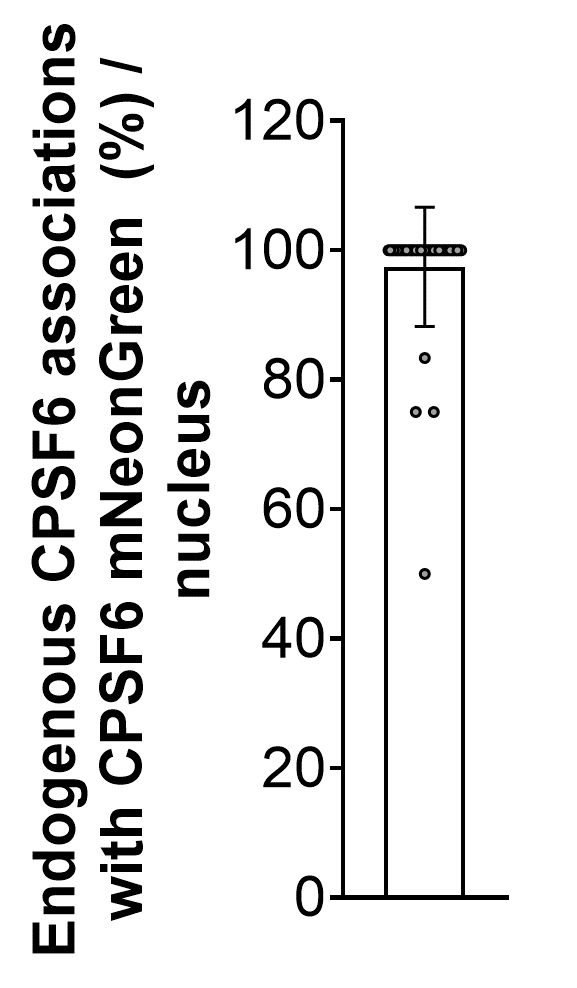


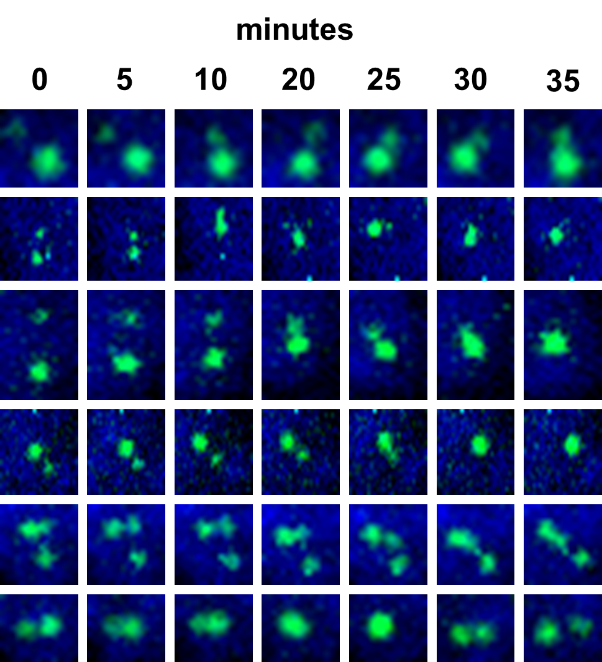

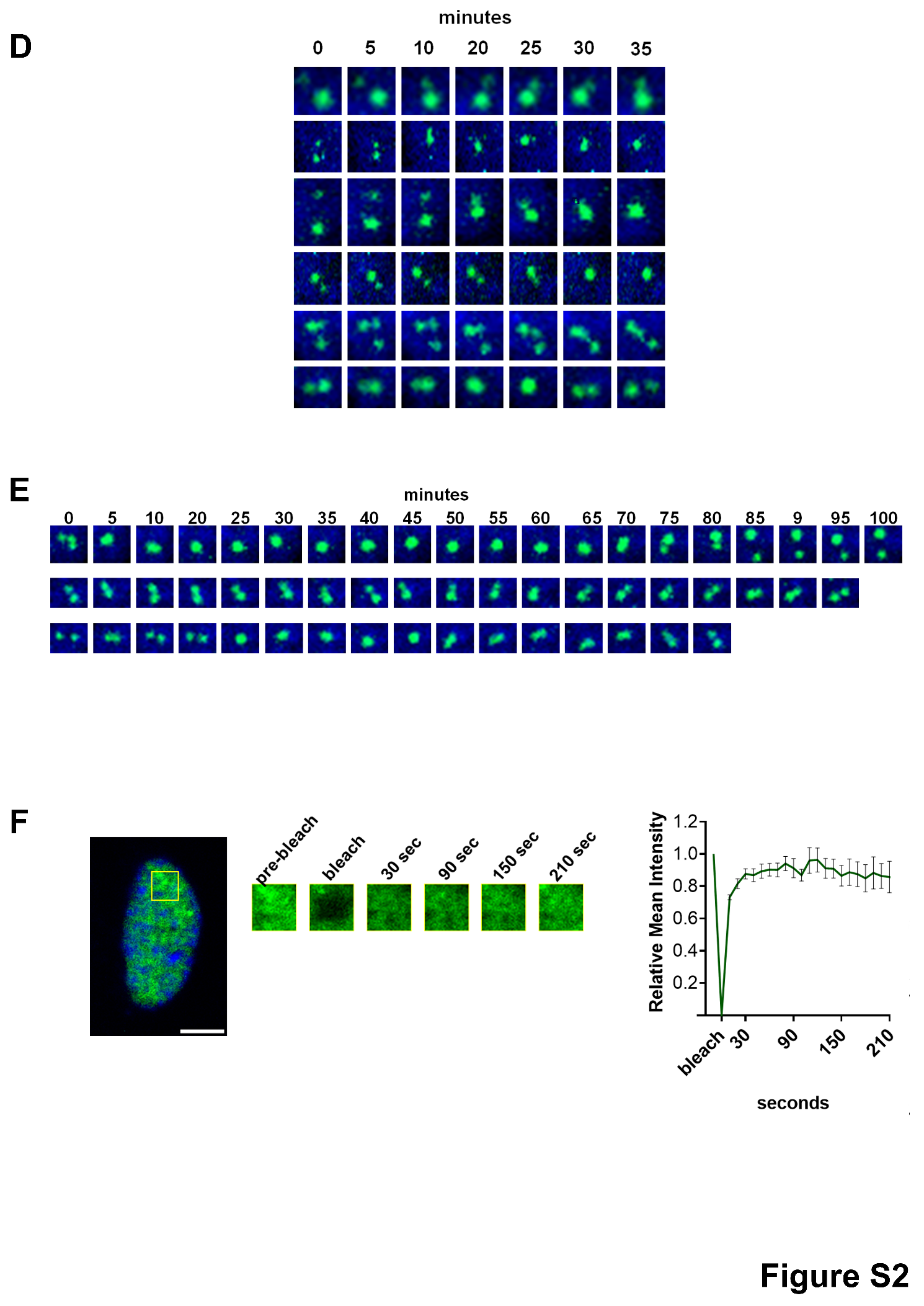


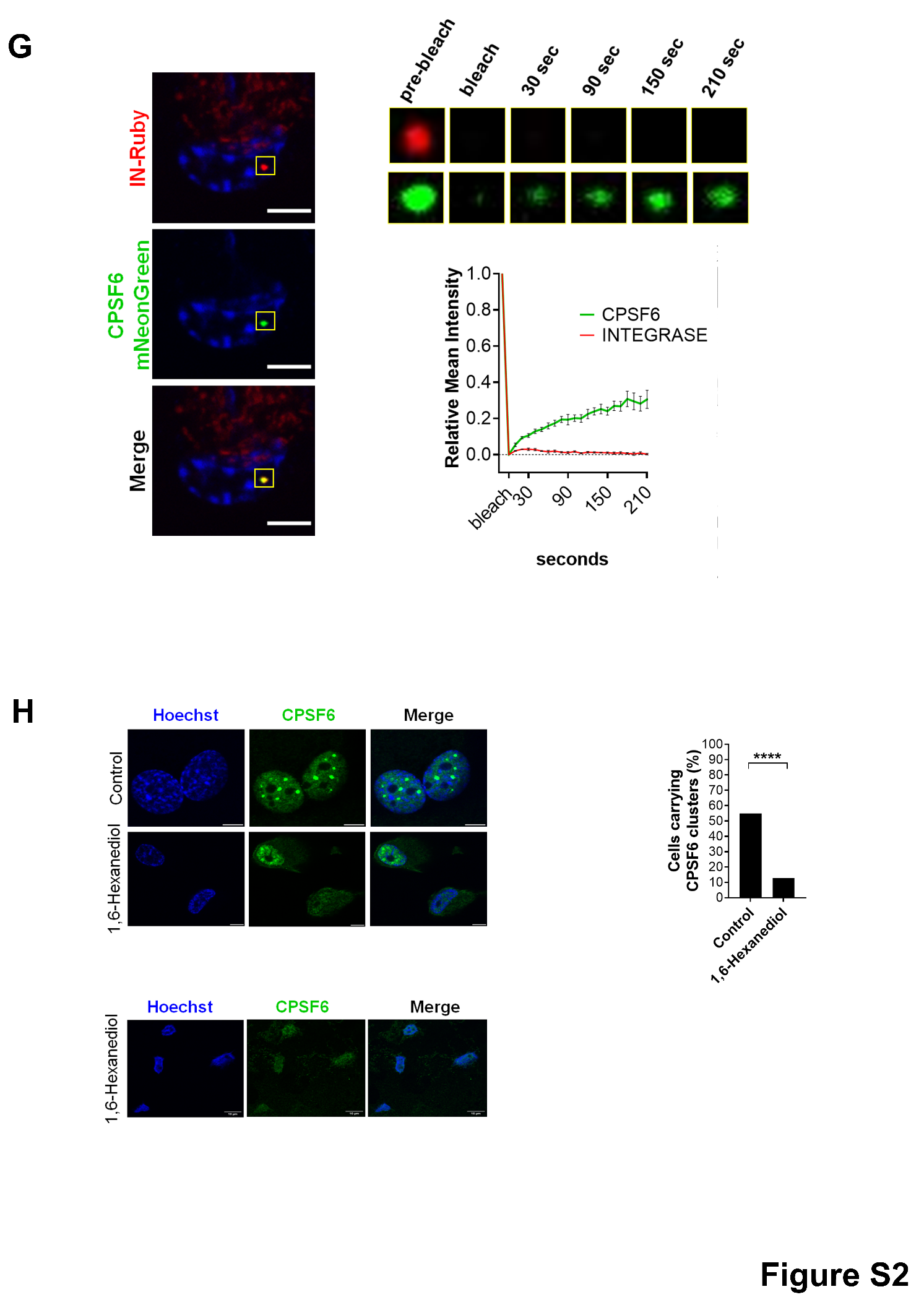


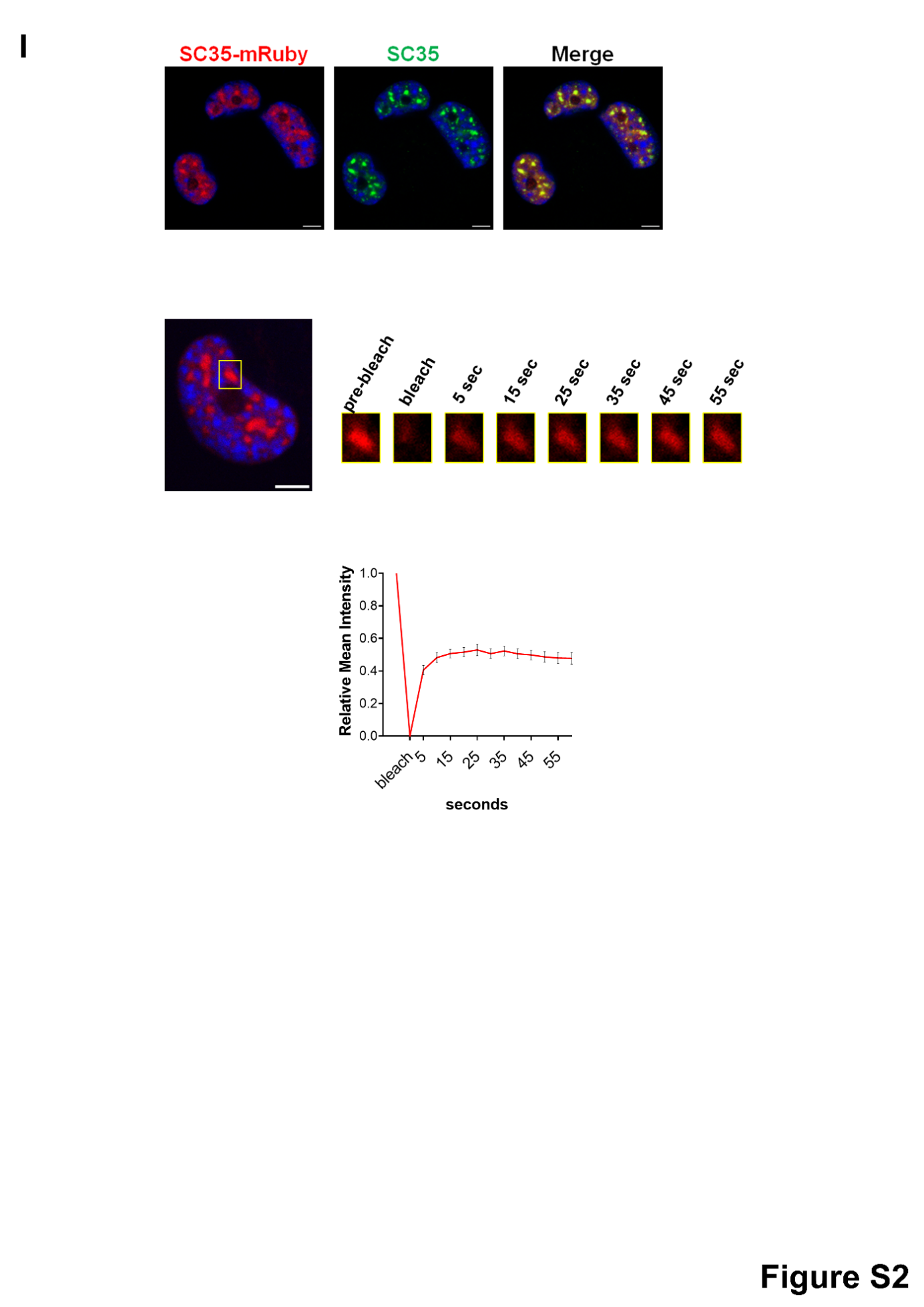


**Supplementary Figure S2. A)** Pearson’s correlation of the mean intensity of CPSF6 clusters with the colocalizing IN focus (****=p ≤ 0.0001) in THP-1 cells infected with HIV-1 (MOI 5) + NEV (10 μM), 24 h and 72 h p.i. 3D analysis. **B)** Confocal microscopy images of THP-1 cells expressing CPSF6 mNeonGreen infected with HIV-1 (MOI 10, 3 days p.i.), stained with anti-CPSF6 antibody (red, above) or with anti-HA (INTEGRASE) (red, below). The graphs show the percentage ± SD of CPSF6 clusters labelled with the antibody that colocalizes with CPSF6 mNeonGreen clusters (n=45 cells), and the percentage ± SD of CPSF6 mNeonGreen clusters colocalizing with IN-HA foci (n=49 cells). **C)** The graph shows the area along the memebraneless organelles fusion-fission event (Figure 2C and Supplementary Video S1). **D)** Short-timing and **E)** Long-timing fusion/fission events from time-lapse microscopy in 2D of THP-1 cells expressing CPSF6 mNeonGreen infected with HIV-1 (MOI 10). Videos acquired 5 min/frame between 24 to 72 h p.i. **F)** Frames extracted form a FRAP time-lapse in uninfected THP-1 cells expressing CPSF6 mNeonGreen. The graph shows the recovery of the signal curve ± SEM (8 FRAP). Pre-bleach signal is set to 1 and bleach signal is set to 0. **G)** Frames extracted form a FRAP time-lapse in THP-1 expressing CPSF6 mNeonGreen infected with HIV-1 GIR (MOI 5, 3 days p.i.). Integrase focus (red) and CPSF6 cluster (green) were in turn bleached with the correspondent laser (561nm/488nm) the foci. The graph shows the recovery of the signal curves ± SEM (19 CPSF6 FRAP, 4 INTEGRASE FRAP). Pre-bleach signal is set to 1 and bleach signal is set to 0. **H)** Confocal microscopy images of THP-1 cells infected with HIV-1 (MOI 5). Twenty-four hours p.i. cells were treated with 10% 1,6-Hexanediol (1,6-Hexa) for 10 minutes. On the right, percentage of cells with CPSF6 clusters (n cells = 60, 55). Fisher’s exact test, ****=p ≤ 0.0001. **I)** On the top confocal microscopy images of THP-1 cells expressing SC35-mRuby (3 days post transduction) stained with anti-SC35 antibody. On the bottom frames extracted form a FRAP time-lapse in uninfected THP-1 cells expressing SC35-mRuby. The graph shows the recovery of the signal curve ± SEM (14 FRAP). Pre-bleach signal is set to 1 and bleach signal is set to 0. Scale bars: 5 μm, unless indicated.


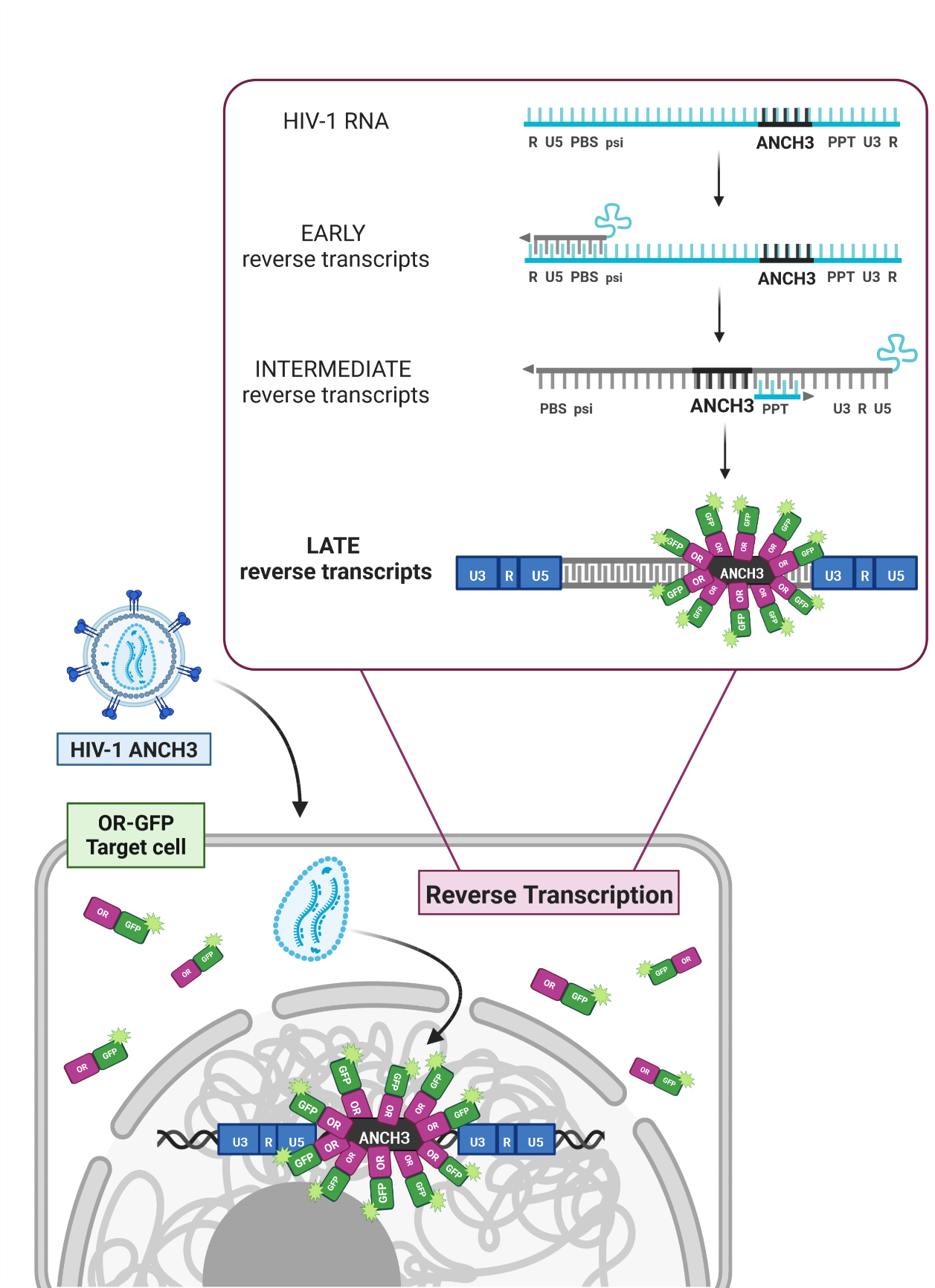


**A**


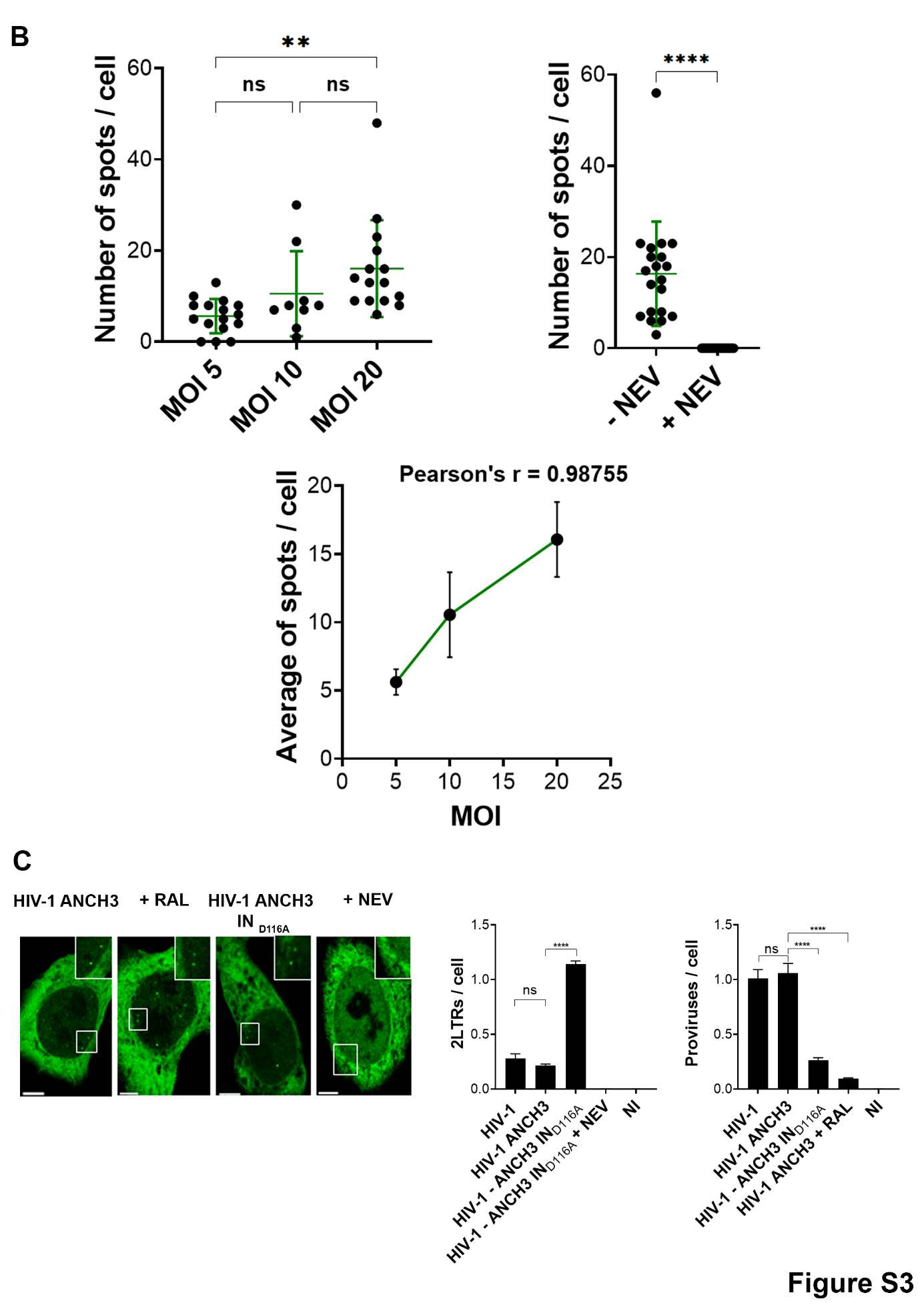


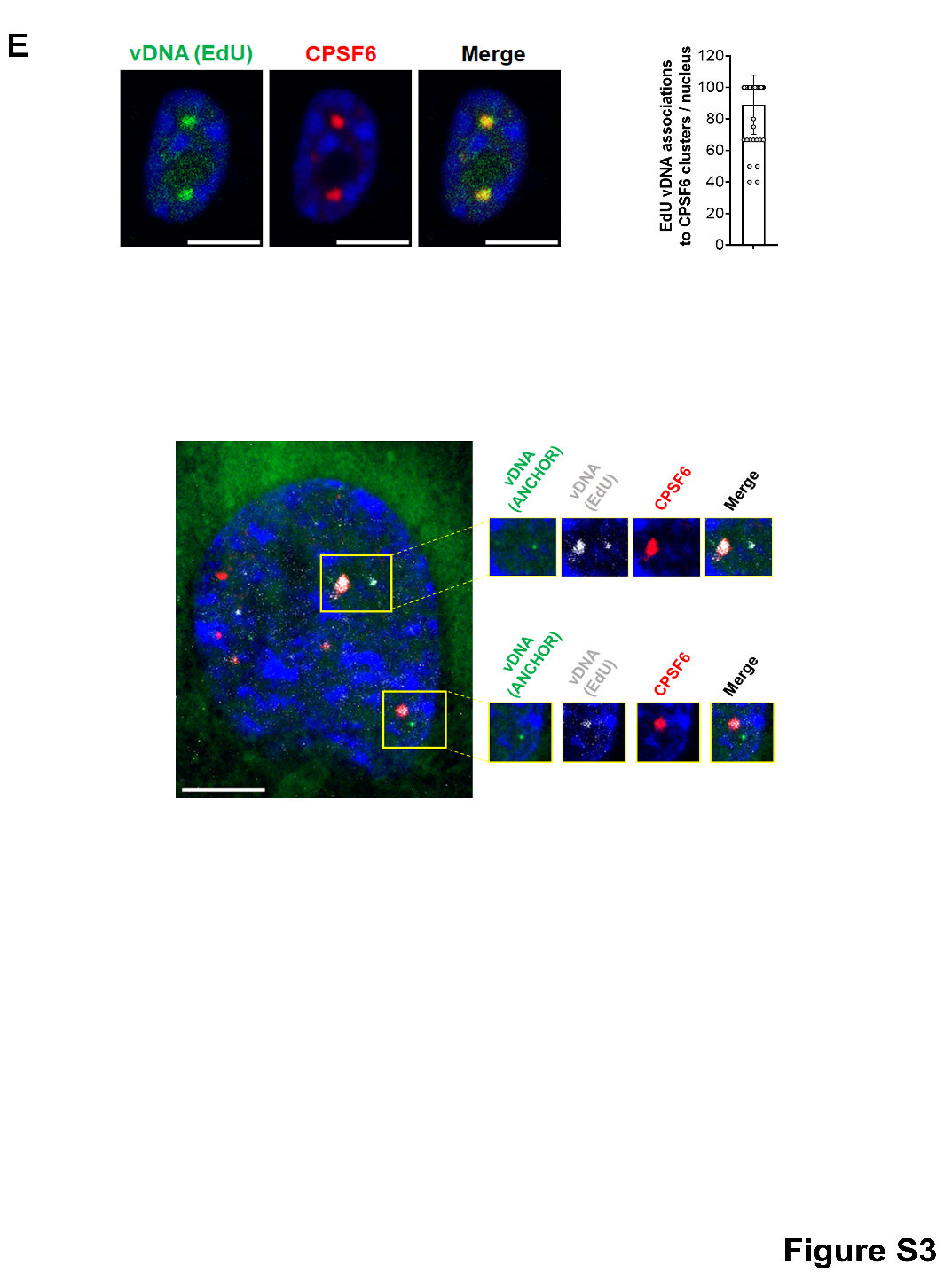

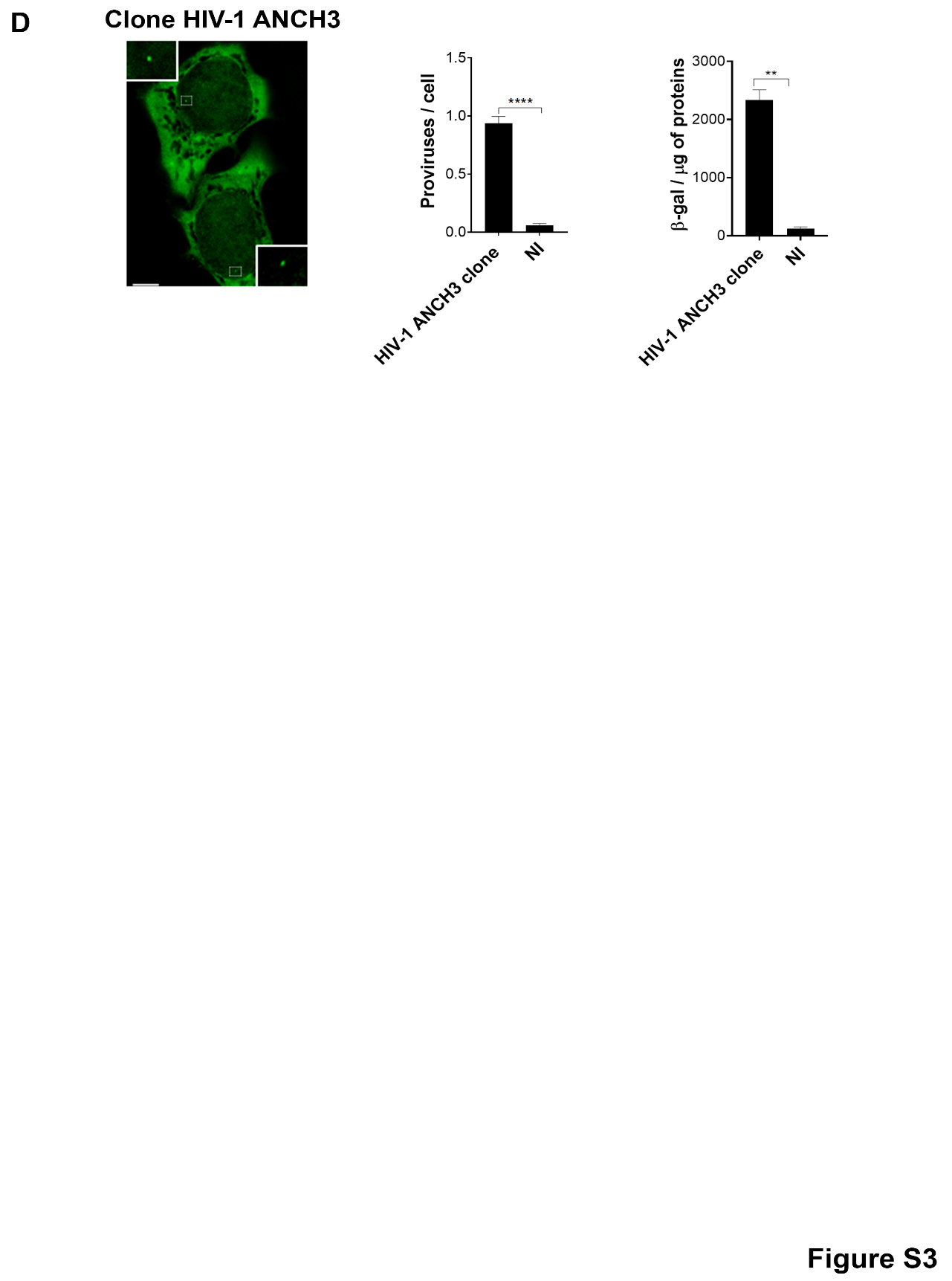

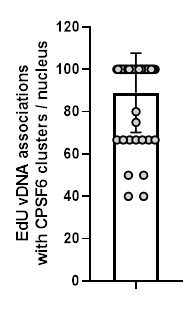


**Supplementary Figure S3. A)** Scheme of HIV-1 ANCHOR system created with BioRender.com. ANCH3 sequence has been cloned in the HIV-1 genome in place of Nef gene (HIV-1 ANCH3). The late retrotranscribed DNA of HIV-1 ANCH3 can be visualized in cells stably expressing the OR-GFP protein, thanks to OR accumulation on the double-stranded ANCH3 sequence. Viral DNA detection occurs mostly in the nucleus, where late reverse transcripts are abundant and are more likely to be exposed to the binding of OR-GFP. **B)** Analysis in HeLa P4R5 cells expressing OR-GFP infected with HIV-1 ANCH3, 24 h p.i. The plots show: the vDNA spots count per cell for each MOI used and the comparison between the count in infected cells treated or not with NEV (10μM). Pearson’s correlation of the average spots count with the increasing MOI used (n cells = 16, 9, 15) ± SEM. **C)** Confocal images of HeLa P4R5 cells expressing OR-GFP infected with HIV-1 ANCH3, HIV-1 ANCH3 + RAL (20 μM), HIV-1 ANCH3 IND116A and HIV-1 ANCH3 + NEV (10 μM) (MOI 30, 24 h p.i.). The histogram plots of 2-LTR circles and ALU-PCR represent the post-nuclear formation of episomal form and integration rates, respectively. One-way ANOVA followed by Tukey’s multiple comparison test, ns=not-significant, ****=p ≤ 0.0001. **D)** Confocal image of HIV-1 ANCH3 provirus in an infected clone. On the right, histogram plot of ALU-PCR and β-galactosidase expression in the infected clone compared to uninfected cells (NI). Unpaired t test, **=p ≤ 0.01, ****=p ≤ 0.0001. **E)** Confocal microscopy images of THP-1 cells infected with HIV-1 in presence of EdU (MOI 20, 3 days p.i.) and quantification of EdU foci associated with CPSF6 clusters per nucleus (n=45 cells). The image below shows a confocal microscopy image of THP-1 infected with HIV-1 ANCH3 in presence of EdU (MOI 20, 3 days p.i.). Scale bars: 5 μm.


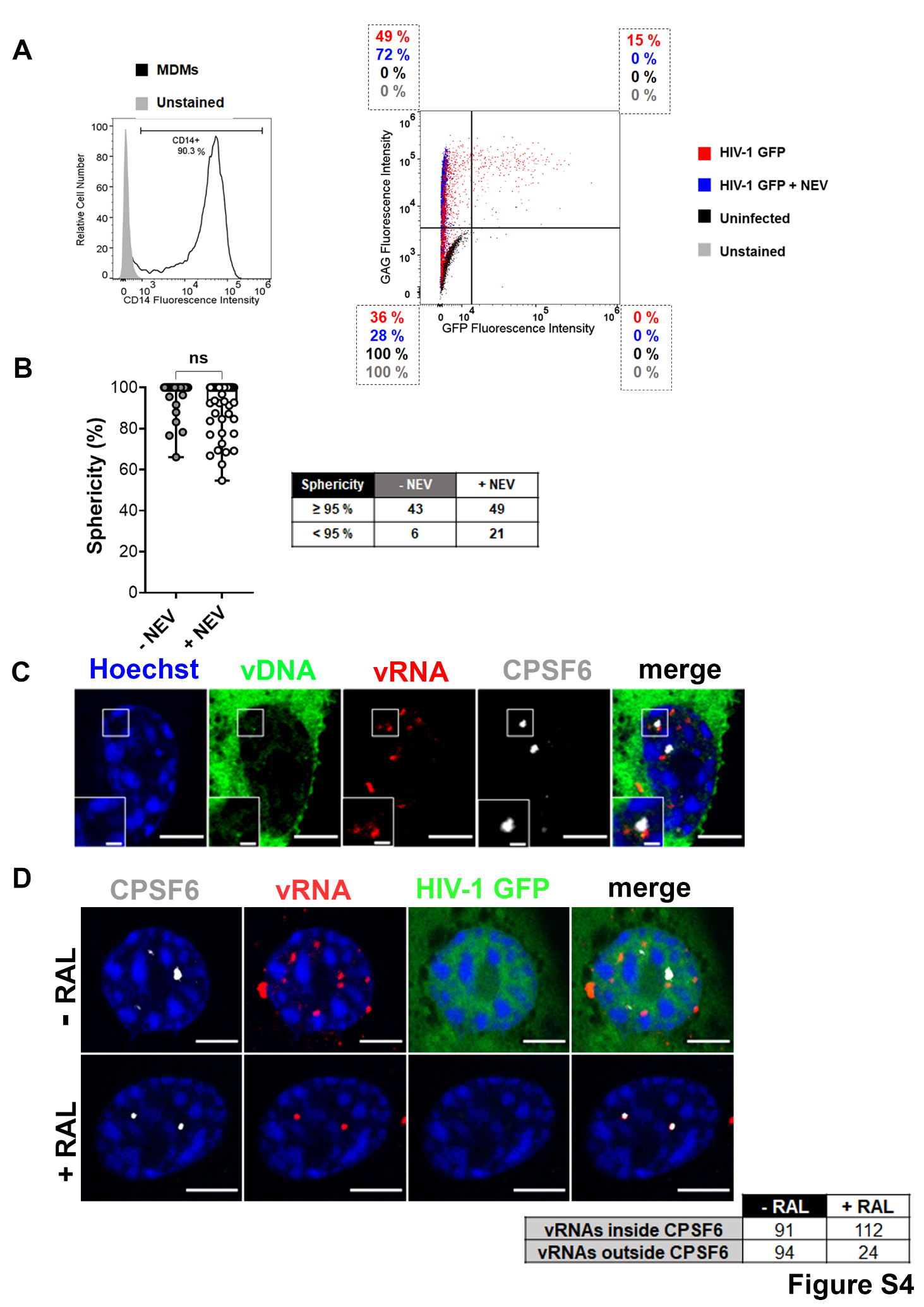


**Supplementary Figure S4. A)** The FACS histogram displays CD14+ expression of the MDMs in this study. The FACS dot plot shows the correlation of GAG signal and GFP expression, with the related percentages per population (MOI 20, 2-3 days p.i.). **B)** Box plot of the sphericity of CPSF6 clusters in infected MDMs. Unpaired t test, ns=not-significant. **C)** Confocal image of Immuno-RNA FISH in THP-1 cell expressing OR-GFP infected with HIV-1 ANCH3 (MOI 20, 3 days p.i.). **D)** Confocal images of Immuno-RNA FISH in THP-1 cells infected with HIV-1 GFP ± RAL (20 μM) (MOI 10, 2 days p.i.). Scale bar: 5 μm, inset: 1 μm.


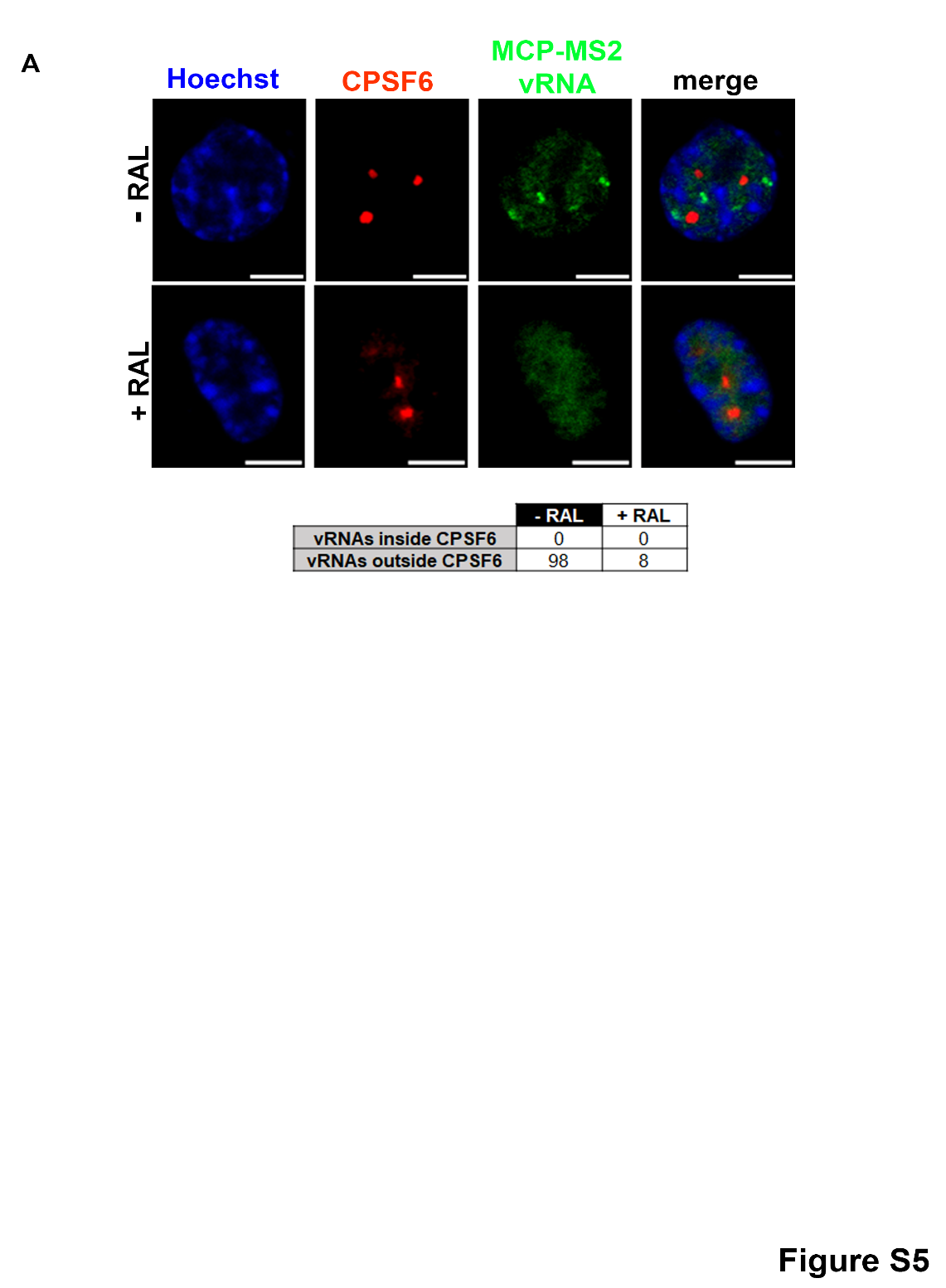

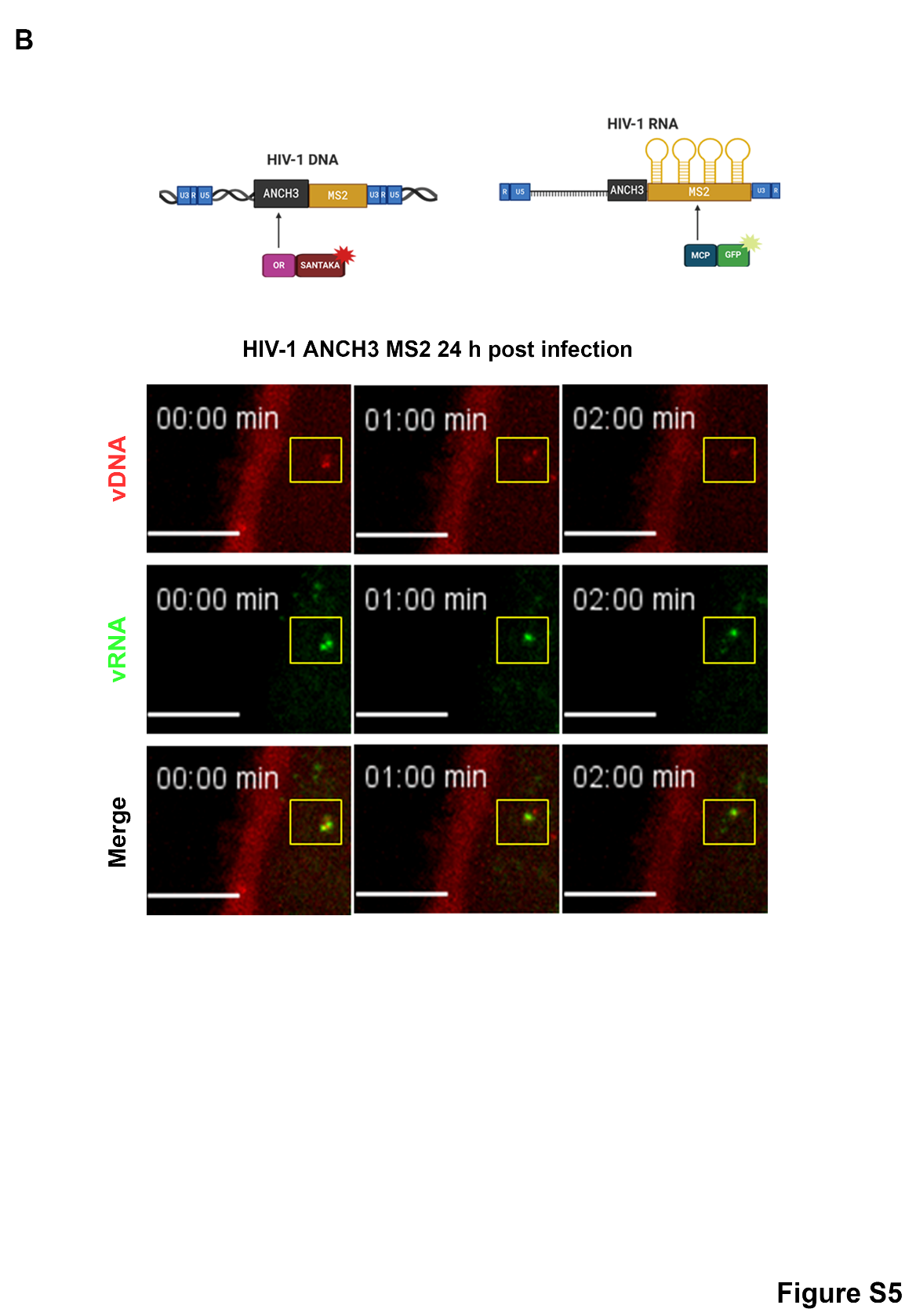


**Supplementary Figure S5. A)** Confocal images of THP-1 cells expressing MCP-GFP infected with HIV-1 ANCH3 MS2 ± RAL (20μM), (MOI 2.5, 3 days p.i.). **B)** The cartoon model shows the double labelling system: the HIV-1 genome is tagged both with ANCH3 and MS2 sequences. The differently fluorescent reporters allow the simultaneous detection of vDNA through the binding of OR-GFP to ANCH3 sequence and of vRNA through the binding of MCP to MS2 stem loops. Created with BioRender.com. Frames from a time-lapse microscopy of HeLa MCP-GFP cells transduced with OR-SANTAKA LV (MOI 1) and 48 hours after infected with HIV-1 ANCH3 MS2 (MOI 50), 24 h p.i.. Projection of 6 Z slices, spacing 0.2 μm with Ti2E inverted microscope (Nikon), based on a CSU-W1 spinning-disk (Yokogawa), using a 60X objective (Plan Apochromat, oil immersion, NA=1.4). Scale bar: 5 μm.


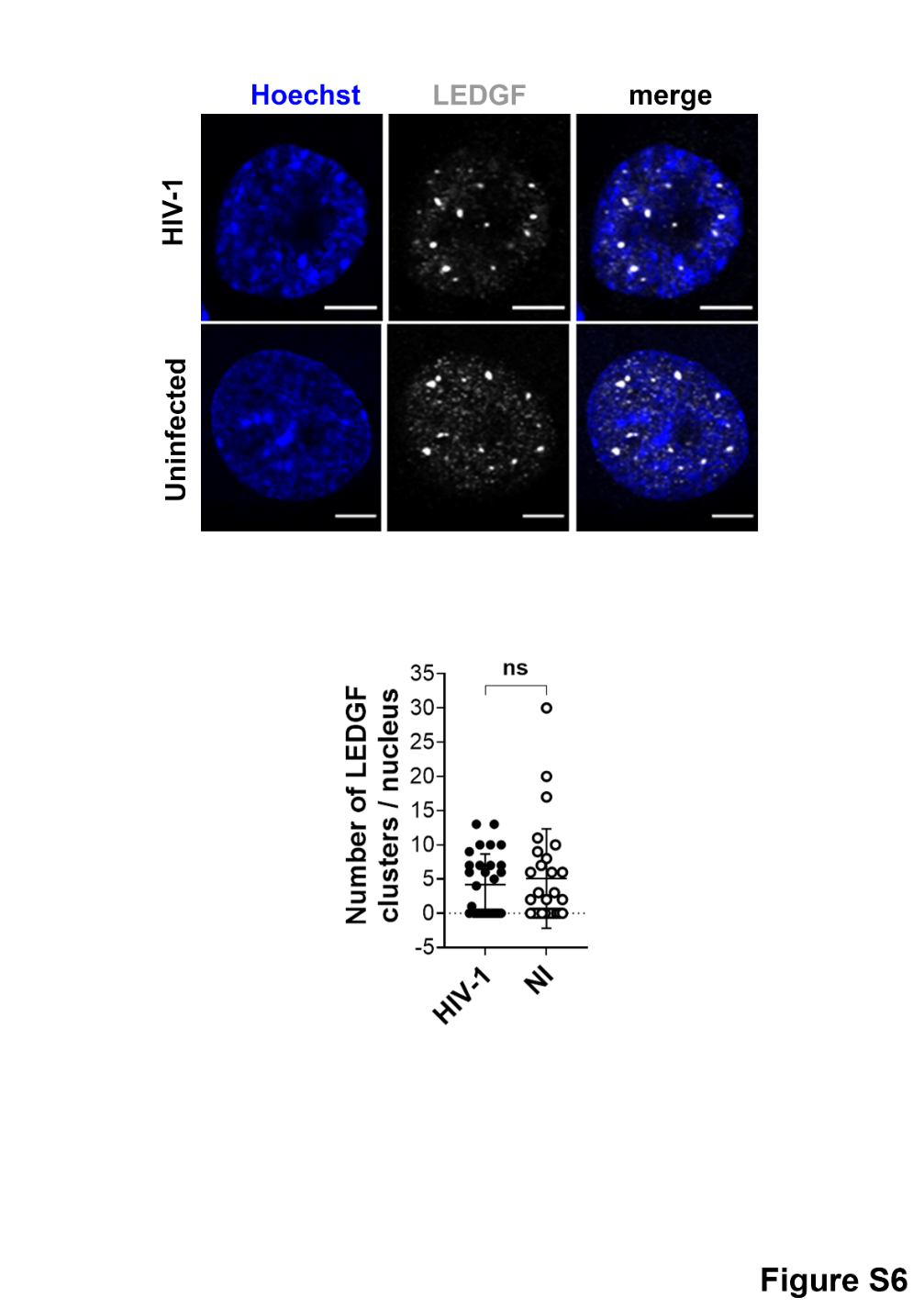


**Supplementary Figure S6. A)** Confocal images of THP-1 cells infected with HIV-1 (MOI10, 3 days p.i.) compared to uninfected cells. The scatter plot shows the count of LEDGF clusters per nucleus (n = 29 cells (HIV-1), 28 cells (NI)); unpaired t test, ns = not-significant, Scale bar: 5 μm.

**Supplementary Video Legends**

**Supplementary Video S1. CPSF6 clusters dynamics in HIV-1 infected cells.** Time-lapse microscopy of THP-1 cells expressing CPSF6 mNeonGreen infected with HIV-1 (MOI 10). Time post infection is indicated on the top. It is possible to appreciate different fusion/fission events of CPSF6 clusters (green). Representative of 6 independent experiments. Scale bar: 5 μm.

**Supplementary Video S2. FRAP time lapse of CPSF6 clusters in HIV-1 infected cells.** FRAP time-lapse in THP-1 cells expressing CPSF6 mNeonGreen infected with HIV-1 (MOI 10, 3 days p.i.). The first 3 frames are pre-bleaching, frame 4 is the bleach and from frame 5 it starts the recovery. Representative of 5 independent experiments. Scale bar: 5 μm.

**Supplementary Video S3. Generation and separation of ds vDNA from the IN focus in live.** Time-lapse microscopy in THP-1 cells infected with HIV-1 ANCH3 GIR virus (MOI 30, 80 h p.i.). The vDNA (green) signal generates from the integrase (IN) proteins focus (red). The video is a montage of continuous 2D frames (5 min/frame). Scale bar: 5 μm.

**Supplementary Methods**

| **Probe Name** | **Sequence** |
| --- | --- |
| HIV1-01 | GGG GAT TGT AGG GAA TTC CAA ATT CCT GCT TTT ACA CTC GGA CCT CGT CGA CAT GCA TT |
| HIV1-02 | CTT TTA GCT GAC ATT TAT CAC AGC TGG CTA TTA CAC TCG GAC CTC GTC GAC ATG CAT T |
| HIV1-03 | GTG TGC TGG TAC CCA TGC CAG ATA GAC TTA CAC TCG GAC CTC GTC GAC ATG CAT T |
| HIV1-04 | AAT ACT GGA GTA TTG TAT GGA TTT TCA GGC CCT TAC ACT CGG ACC TCG TCG ACA TGC ATT |
| HIV1-05 | TTT TAC TGG TAC AGT CTC AAT AGG GCT AAT GGT TAC ACT CGG ACC TCG TCG ACA TGC ATT |
| HIV1-06 | TAT GTT GAC AGG TGT AGG TCC TAC TAA TAC TGT TAC ACT CGG ACC TCG TCG ACA TGC ATT |
| HIV1-07 | CTA ATC CTC ATC CTG TCT ACT TGC CAT TAC ACT CGG ACC TCG TCG ACA TGC ATT |
| HIV1-08 | CAA TCA TCA CCT GCC ATC TGT TTT CCA TTT ACA CTC GGA CCT CGT CGA CAT GCA TT |
| HIV1-09 | TTT CCA AAG TGG ATT TCT GCT GTC CCT GTA TTA CAC TCG GAC CTC GTC GAC ATG CAT T |
| HIV1-10 | TTG TGG ATG AAT ACT GCC ATT TGT ACT GCT GTT ACA CTC GGA CCT CGT CGA CAT GCA TT |
| HIV1-11 | TTA AGA TGT TCA GCC TGA TCT CTT ACC TGT TTA CAC TCG GAC CTC GTC GAC ATG CAT T |
| HIV1-12 | TAC AGT CTA CTT GTC CAT GCA TGG CTT CTT ACA CTC GGA CCT CGT CGA CAT GCA TT |
| HIV1-13 | TCA TGT TCA TCT TGG GCC TTA TCT ATT CCT TAC ACT CGG ACC TCG TCG ACA TGC ATT |
| HIV1-14 | TGT CAG TTA GGG TGA CAA CTT TTT GTC TTC CTT TAC ACT CGG ACC TCG TCG ACA TGC ATT |
| HIV1-15 | TGC TCC TAC TAT GGG TTC TTT CTC TAA CTT TAC ACT CGG ACC TCG TCG ACA TGC ATT |
| HIV1-16 | TCT GTT AGT GCT TTG GTT CCT CTA AGG AGT TTT TAC ACT CGG ACC TCG TCG ACA TGC ATT |
| HIV1-17 | CTG TAT GTC ATT GAC AGT CCA GCT GTC TTT TTT ACA CTC GGA CCT CGT CGA CAT GCA TT |
| HIV1-18 | TGG CAG CAC TAT AGG CTG TAC TGT CCT TAC ACT CGG ACC TCG TCG ACA TGC ATT |
| HIV1-19 | TCT GAT GTT TTT TGT CTG GTG TGG TAA GTC CCT TAC ACT CGG ACC TCG TCG ACA TGC ATT |
| HIV1-20 | CCT CAA CAG ATG TTG TCT CAG CTC CTC TTA CAC TCG GAC CTC GTC GAC ATG CAT T |
| HIV1-21 | ATT GCT GGT GAT CCT TTC CAT CCC TGT TAC ACT CGG ACC TCG TCG ACA TGC ATT |
| HIV1-22 | TTT CTT TTT TAA CCC TGC GGG ATG TGG TAT TCT TAC ACT CGG ACC TCG TCG ACA TGC ATT |
| HIV1-23 | TTT AAC TTT TGG GCC ATC CAT TCC TGG CTT ACA CTC GGA CCT CGT CGA CAT GCA TT |
| HIV1-24 | CCC TAT CTT TAT TGT GAC GAG GGG TCG TTG TTA CAC TCG GAC CTC GTC GAC ATG CAT T |

**Supplementary Table S1. Probes used for RNA FISH against pol**

**Quantitative PCR and primers**

For HIV-1 ANCH3 DNA forms quantification in HeLa P4R5 cells, a total DNA of 10^6^ infected cells (MOI 30) was extracted at 6 hours post infection for Late Reverse Transcripts (LRTs) qPCR and 24 hours post infection for 2LTR-circles and ALU-PCR, through QIAmp DNA micro kit (QIAGEN #56304). Real-Time PCR of LRTs was performed to assess DNA synthesis and used to normalize qPCR data on viral input, the reactions were carried on in 20 µL, in iTaqUniversal SYBR Green Supermix (Bio-Rad #1725124) using primers for U3 sequence: U3 FX: 5’-TTCCGCTGGGGACTTTCCAGGG-3’, U3 RX: 5’-AGGCTCAGATCTGGTCTAACC-3’. Real-Time PCR of 2LTR-circles was used to assess nuclear import efficiency. Reactions were performed in 20 µL, in Maxima Probe/ROX qPCR Mastermix (ThermoFisher #K0232) using primers for 2LTR-circle junction: 2LTR FX: 5’-AACTAGGGAACCCACTGCTTAAG-3’, 2LTR RX: 5′-TCCACAGATCAAGGATATCTTGTC-3′, 2-LTR probe: 5’-(FAM)-ACACTACTTGAAGCACTCAAG-GCAAGCTTT-(TAMRA)-3’. Proviral integration quantification of HeLa P4R5 infected cells and HIV-1 ANCH3 clone was performed through ALU PCR, consisting in a first non-kinetic PCR step in 50 µL using Platinum SuperFi DNA Polymerase kit (ThermoFisher #12351250) for the amplification of ALU-U3 fragments and in a second step of HIV-1 specific qPCR reaction in 20 µL in Maxima Probe/ROX qPCR Mastermix (ThermoFisher #K0232). Primer Alu 166: 5’-TCCCAGCTACTCGGGAGGCTGAGG-3’, Alu 2: 5’-GCCTCCCAAAGTGCTGGGATTACAG-3’, LambdaU3: 5′-ATGCCACGTAAGCGAAACTTTCCGCTGGGGACTTTCCAGGG-3′ for ALU PCR. Lambda: 5’-ATGCCACGTAAGCGAAACT-3’, U5: 5’-CTGACTAAAAGGGTCTGAGG-3’, Probe: 5’-(FAM)- TTAAGCCTCAATAAAGCTTGCCTTGAGTGC-(TAMRA) for qPCR. In all experiments β-actin detection was used for normalization. β-actin FX: 5’-AACACCCCAGCCATGTACGT-3’, β-actin RX: 5-CGGTGAGGATCTTCATGAGGTAGT-3’, β-actin probe: (FAM)-CCAGCCAGGTCCAGACGCAGGA-(BHQ1).
